# Multi-omics analysis identifies a liquid-liquid phase separation-related subtypes in head and neck squamous cell carcinoma

**DOI:** 10.3389/fonc.2025.1509810

**Published:** 2025-02-26

**Authors:** Peng-Lei Zhai, Meng-Min Chen, Qi Wang, Jing-Jun Zhao, Xiao-Mei Tang, Cui-Ni Lu, Jia Liu, Qin-Xin Yang, Ming-Liang Xiang, Qing-Hai Tang, Biao Gu, Shu-Ping Zhang, Si-Ping Tang, Da Fu

**Affiliations:** ^1^ Key Laboratory of Functional Organometallic Materials of College of Hunan Province, College of Chemistry and Materials Science, Hengyang Normal University, Hengyang, China; ^2^ Department of General Surgery, Pancreatic Disease Center, Ruijin Hospital, Shanghai Jiao Tong University School of Medicine, Shanghai, China; ^3^ Research Institute of Pancreatic Diseases, Shanghai Key Laboratory of Translational Research for Pancreatic Neoplasms, Shanghai Jiao Tong University School of Medicine, Shanghai, China; ^4^ State Key Laboratory of Oncogenes and Related Genes, Institute of Translational Medicine, Shanghai Jiao Tong University, Shanghai, China; ^5^ Department of Urology, Ruijin Hospital, Shanghai Jiao Tong University School of Medicine, Shanghai, China; ^6^ Department of Otolaryngology & Head and Neck Surgery, Ruijin Hospital, Shanghai Jiao Tong University School of Medicine, Shanghai, China; ^7^ Hunan Key Laboratory for Conservation and Utilization of Biological Resources in the Nanyue Mountainous Region, College of Life Sciences, Hengyang Normal University, Hengyang, China

**Keywords:** liquid-liquid phase separation, genomic alterations, tumor immune microenvironment, immunotherapy, drug prediction

## Abstract

**Background:**

Growing evidence indicates that abnormal liquid–liquid phase separation (LLPS) can disrupt biomolecular condensates, contributing to cancer development and progression. However, the influence of LLPS on the prognosis of head and neck squamous cell carcinoma (HNSCC) patients and its effects on the tumor immune microenvironment (TIME) are not yet fully understood. Therefore, we aimed to categorize patients with HNSCC based on LLPS-related genes and explored their multidimensional heterogeneity.

**Methods:**

We integrated the transcriptomic data of 3,541 LLPS-related genes to assess the LLPS patterns in 501 patients with HNSCC within The Cancer Genome Atlas cohort. Subsequently, we explored the differences among the three LLPS subtypes using multi-omics analysis. We also developed an LLPS-related prognostic risk signature (LPRS) to facilitate personalized and integrative assessments and then screened and validated potential therapeutic small molecule compounds targeting HNSCC via experimental analyses.

**Result:**

By analyzing the expression profiles of 85 scaffolds, 355 regulators, and 3,101 clients of LLPS in HNSCC, we identified three distinct LLPS subtypes: LS1, LS2, and LS3. We confirmed notable differences among these subtypes in terms of prognosis, functional enrichment, genomic alterations, TIME patterns, and responses to immunotherapy. Additionally, we developed the LPRS, a prognostic signature for personalized integrative assessments, which demonstrated strong predictive capability for HNSCC prognosis across multiple cohorts. The LPRS also showed significant correlations with the clinicopathological features and TIME patterns in HNSCC patients. Furthermore, the LPRS effectively predicted responses to immune checkpoint inhibitor therapy and facilitated the screening of potential small-molecule compounds for treating HNSCC patients.

**Conclusion:**

This study presents a new classification system for HNSCC patients grounded in LLPS. The LPRS developed in this research offers improved personalized prognosis and could optimize immunotherapy strategies for HNSCC.

## Introduction

Head and neck squamous cell carcinoma (HNSCC) is the sixth most prevalent cancer globally and the most common malignancy in the head and neck region, primarily arising from the mucosal epithelium of the oral cavity, pharynx, and larynx (1). HNSCC significantly contributes to cancer-related morbidity and mortality, with risk factors including tobacco use, alcohol consumption, and human papillomavirus (HPV) infection (2). Over 70% of HNSCC cases are diagnosed at a locally or regionally advanced stage, and 10% present with distant metastases (3). The 5-year overall survival (OS) rate for locally advanced HNSCC remains under 50%, and it drops to just 5% for recurrent or metastatic cases (4). Thus, identifying biomarkers that can improve patient prognosis and facilitate targeted treatment is critical.

Liquid–liquid phase separation (LLPS) occurs when biological macromolecules, such as proteins or nucleic acids, form droplet-like condensates without an enclosing membrane through weak multivalent interactions (5). LLPS underlies the formation of various membraneless organelles, including stress granules, processing bodies, and nuclear speckles (6). This process allows cells to efficiently conduct biological activities by compartmentalizing specific macromolecules within membraneless spaces, facilitating functions such as chromatin remodeling and regulation of gene transcription and translation (7). Research indicates that LLPS plays a significant role in the development and treatment of human diseases, including cancer (8). For instance, in colon cancer, the phase-separated DDX21 protein activates MCM5, which triggers epithelial–mesenchymal transition (EMT) signaling and promotes metastasis.

However, disrupting phase separation reduces MCM5 activation, thereby hindering metastasis (9). Similarly, interfering with the phase separation of YAP, a key protein in tumor progression, can suppress cancer cell proliferation and enhance immune responses (10). It is important to note that a significant portion of the malignant properties of tumors is linked to intrinsically disordered domains (IDRs) in proteins (11), which are regulated by LLPS (12, 13). Recent studies have shown that CYTOR interacts with FOSL1 to form phase-separated condensates, which can activate FOSL1-dependent super-enhancers (SEs) to promote tumorigenesis and metastasis in head and neck squamous cell carcinoma (HNSCC) (14). LLPS also interacts with factors related to the cytoskeleton, intercellular adhesion molecules, and matrix degradation, affecting tumor cell morphology and migratory abilities. By influencing the phase separation process of these factors, tumor cells may acquire enhanced migration and invasion capabilities, thereby promoting metastasis (15). Additionally, LLPS subtypes may impact immune therapy responses. For example, in bladder cancer, LLPS subtypes have been identified with distinct immune features, which could guide targeted therapies (16). These findings underscore the potential role of LLPS in cancer. Understanding how LLPS influences cancer can deepen our insight into its underlying pathological mechanisms and aid in prognosis and selecting personalized treatments. However, in the context of HNSCC, research on the link between LLPS and distant metastasis remains limited.

**Figure 1:**
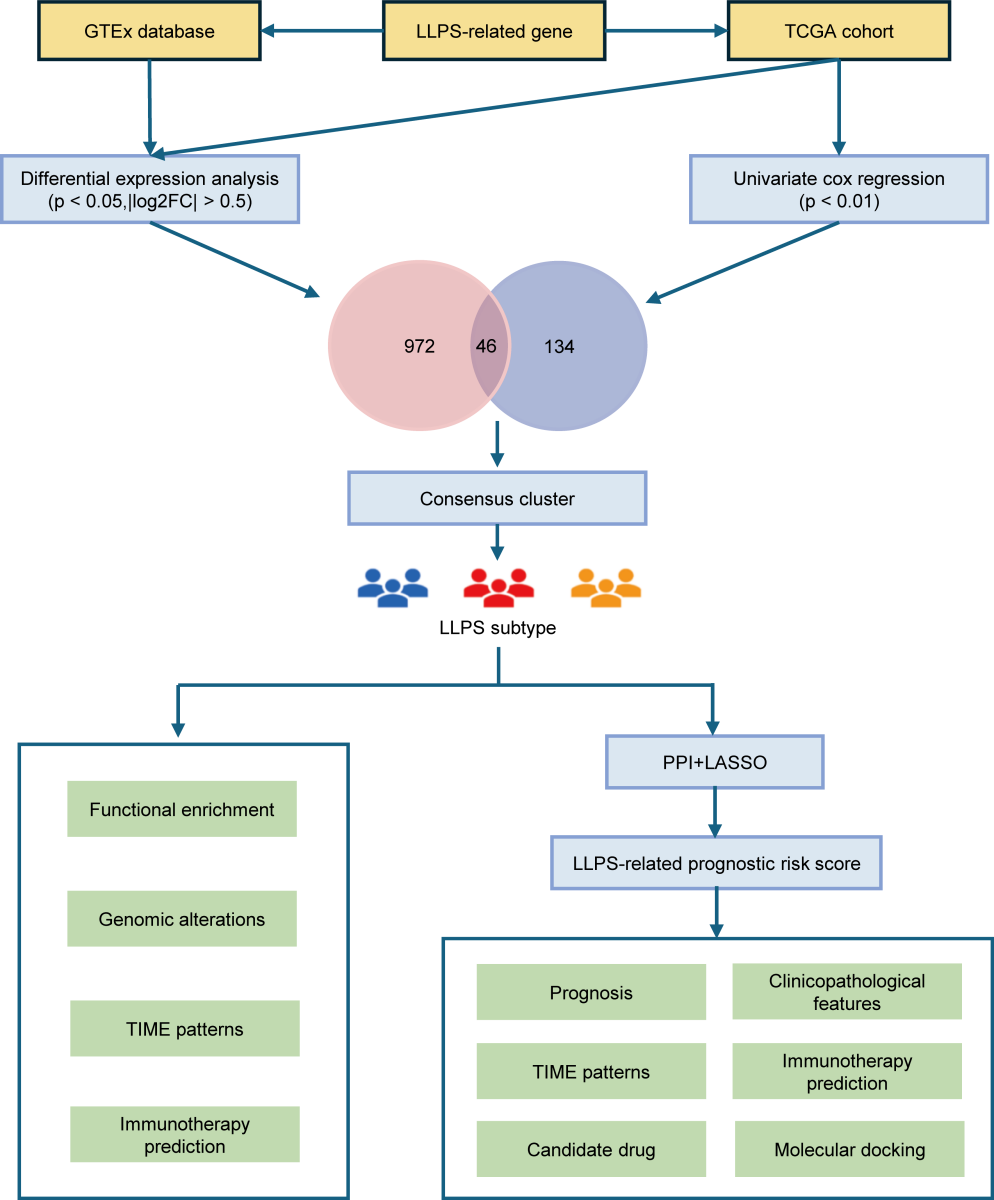
The overall flow diagram of this study.

Therefore, in this study, we utilized The Cancer Genome Atlas (TCGA) dataset to categorize patients with HNSCC based on LLPS-related genes and explored their multidimensional heterogeneity. We also evaluated the prognostic significance of these LLPS-related genes in HNSCC, developing the LLPS-related prognostic signature (LPRS). Finally, we assessed the potential of this signature to predict patient outcomes and responsiveness to immune checkpoint inhibitor (ICI) therapy (**Figure 1**).

## Materials and methods

### Data acquisition and processing

RNA sequencing (RNA-seq) data and clinical information for HNSCC patients were sourced from the TCGA (https://portal.gdc.cancer.gov/), Gene Expression Omnibus (GEO, http://www.ncbi.nlm.nih.gov/geo/), and International Cancer Genome Consortium (ICGC; https://dcc.icgc.org/) databases. Additionally, RNA-seq data for 366 normal tissues were retrieved from the Genotype-Tissue Expression (GTEx) Portal (https://gtexportal.org/home/). Patients who had incomplete survival data, an OS of less than 30 days, or lacked a definitive histopathological diagnosis were excluded from the study. Three cohorts were compiled for analysis: TCGA-HNSC (N = 501), GSE41613 (N = 97), and ICGC-HNSC (N = 161).

The DrLLPS database (http://llps.biocuckoo.cn/) was used as a comprehensive resource for LLPS-related proteins, documenting 150 scaffolds, 987 regulators, and 8144 clients, all experimentally identified in various eukaryotic species. Our study focused specifically on *Homo sapiens*, retaining 3,633 LLPS-related genes after excluding those identified in other species. Of these, 3,541 LLPS-related genes (comprising 85 scaffolds, 355 regulators, and 3,101 clients) with available expression data from the TCGA cohort were selected for further analysis. Furthermore, all expression data were normalized to log_2_ (FPKM+1) to ensure consistency across multiple databases. For the GSE41613 cohort, the “normalizeBetweenArrays” function from the R package “limma” was employed for background correction and quantitative normalization.

### Determination of LLPS subtypes in HNSCC patients

The expression data for LLPS-related genes were analyzed to identify differentially expressed genes (DEGs) between HNSCC and normal tissues (|log_2_FC|>0.5, P<0.05). These DEGs were then subjected to univariate Cox regression to identify LLPS-related prognostic DEGs. Functional annotation was carried out using Gene Ontology (GO) and KEGG pathway analysis with the “clusterProfiler” R package. The prognostic DEGs were clustered using a consensus clustering algorithm to define LLPS subtypes in HNSCC patients. To confirm the reliability of the clustering, the t-distributed stochastic neighbor embedding (tSNE) algorithm was applied. Kaplan-Meier survival curves were employed to the assess survival differences across the LLPS subtypes. Additionally, single-sample gene set enrichment analysis was used to quantify the enrichment of markers for each LLPS subtype, using gene sets from the MSigDB database.

### Genomic alteration analysis of LLPS subtypes

We obtained Mutation Annotation Format (MAF) files from the TCGA database to analyze and visualize the gene mutation profiles of various LLPS subtypes using the “maftools” R package. In addition, copy number alteration (CNA) data for HNSCC patients were also retrieved from the TCGA database. To identify significant genomic amplifications or deletions, the CNA data were formatted into “.seg” files for analysis with the GISTIC2.0 module. The relevant marker file was downloaded from the official TCGA website (https://gdc.cancer.gov/about-data/gdc-data-processing/gdc-reference-files). The analysis was then carried out using the GISTIC tool, accessible on the GenePattern platform (https://cloud.genepattern.org/) (17).

### Evaluation of tumor immune microenvironment and immunotherapy responses

The immune score, stromal score, ESTIMATE score, and tumor purity for HNSCC patients were computed using the ESTIMATE algorithm via the “estimate” R package (18). Additionally, the enrichment scores for 28 immune features were calculated through single-sample Gene Set Enrichment Analysis (ssGSEA) (19). Based on the median ssGSEA Z-scores, patients were categorized into high- or low-immunity subtypes. The CIBERSORT algorithm was employed to assess the composition of 22 immune cell types (20). To gauge the effectiveness of anti-PD1 and anti-CTLA4 therapies in HNSCC patients, the Tumor Immune Dysfunction and Exclusion (TIDE) algorithm (accessible at http://tide.dfci.harvard.edu/) was utilized.

### Construction and evaluation of LPRS

To identify hub genes linked to the prognosis of LLPS subtypes, a protein–protein interaction (PPI) network was constructed using the STRING database (https://www.string-db.org/) for LLPS-subtype-related DEGs (21). This network was then imported into Cytoscape version 3.8 (https://cytoscape.org/) for hub gene analysis (22). PPIs were analyzed using the degree algorithm within the cytoHubba plugin, and hub genes were identified based on their degree scores. A total of 26 LLPS-subtype-related hub genes were identified.

To develop the LLPS-related prognostic signature, least absolute shrinkage and selection operator (LASSO) regression analysis was conducted using the “glmnet” R package, with the model optimized by selecting the minimum λ through ten-fold cross-validation. The LASSO algorithm was employed to enhance the model’s accuracy and robustness. The LPRS was then calculated as follows:


$$LPRS=\sum_{i=1}^{n}x_{i}*coef_{i}$$

Here, xi represents the expression levels of the selected hub genes and coefi corresponds to the respective LASSO coefficient. The prognostic value of the LPRS was assessed using Kaplan-Meier survival curves and log-rank tests across all cohorts. Additionally, receiver operating characteristic (ROC) curves were employed to evaluate the predictive accuracy of LPRS for 1-year, 3-year, and 5-year OS in HNSCC patients.

### LPRS impact across independent ICI therapy cohorts

To validate the role of LPRS in predicting ICI treatment response, our study used the R package “IMvigor210CoreBiologies” to obtain the IMvigor210 cohort, aiming to study the atezolizumab response to anti-PDL1 in metastatic urothelial carcinoma (23). The gene expression profiles of the cohorts were converted into the log_2_ (FPKM+1) format for better comparability. The LPRS was calculated for each patient to assess its correlation with the ICI treatment response.

### Cell culture and transient transfection

Human head and neck squamous cell carcinoma (HNSCC) cell lines, FaDu (RRID: CVCL_1218) and Detroit562 (RRID: CVCL_1171), were sourced from the Cell Bank of the Chinese Academy of Sciences. Additionally, Cal27 (RRID: CVCL_1107) and HN6 (RRID: CVCL_8129) cell lines were acquired from Bioegene (Shanghai, China). All mentioned cell lines underwent authentication via STR profiling. The cells were cultured in Dulbecco’s Modified Eagle Medium (DMEM, catalog number: MA0212, MeilunBio, Shanghai, China) supplemented with 10% Fetal Bovine Serum (FBS, catalog number: S1001-500, BIOAGRIO, Brazil) and 1% Penicillin/Streptomycin (P/S, catalog number: C100C5, NCM Biotech, Suzhou, China), and were maintained at 37°C in an atmosphere containing 5% CO_2_. To ensure the absence of mycoplasma contamination, all cell lines were routinely treated with mycoplasma removal agents (Biosharp, catalog number: BL591B, Hefei, China). Transfection of NT5E siRNA (Genechem, Shanghai, China) was performed using Lipofectamine 2000 (Invitrogen, catalog number: 11668030, Carlsbad, CA). The target sequence of NT5E siRNA was GGGTGTATACTGTGAGATCAA (NT5E-si).

### RNA extraction and real-time quantitative PCR

RNA primers were synthesized by Sangon Biotech (Shanghai, China). Total RNA from cells and tissues was extracted using the FastPure^®^ Cell/Tissue Total RNA Isolation Kit V2 (Vazyme Biotech, catalog number: RC101-01, Nanjing, China). Reverse transcription was performed using the HiScript III All-in-one RT SuperMix for qPCR (Vazyme Biotech, catalog number:R333-01, Nanjing, China). RT-qPCR detection of NT5E expression levels was performed according to the instructions of the ChamQ SYBR qPCR Master Mix (Vazyme Biotech, catalog number: Q711-02, Nanjing, China). The primers used are listed in **Supplementary Table S1**.

### Fluorescence recovery after photobleaching assay

FRAP experiments were performed using a Zeiss LSM900 confocal microscope at 37°C with 5% CO2. A 488 nm laser at 30% maximum power was used to bleach the fluorescence signal within a region of 1–2 µm diameter for 10 seconds. Fluorescence recovery was monitored at a rate of 2 seconds per frame. For image acquisition, intensities were corrected for global photobleaching by subtracting background signals and adjusting for fluorescence from a nearby unbleached droplet.

### Cell viability assay

The evaluation of cell proliferation ability and the IC50 of drugs were conducted using the Cell Counting Kit-8 (CCK-8; MeilunBio, catalog number: MA0218-1, Dalian, China). Cells in the logarithmic growth phase were digested with 0.25% trypsin. A single-cell suspension was prepared in medium containing 10% Fetal Bovine Serum (FBS). Cells were seeded at a working density of 5000 cells per well in a 96-well plate and cultured at 37°C in a humidified atmosphere with 5% CO_2_ until adhesion. Subsequently, at different time points, the supernatant was discarded, and each well was supplemented with 10 μL of CCK-8 solution and 90 μL of culture medium. The absorbance at 450 nm was measured after 2 hours.

### Colony formation assay

Cells were seeded at a density of 500 cells per well in a 6-well plate and maintained in normal medium for two weeks, with medium replaced every two days. Subsequently, the cells were fixed and stained with 0.1% crystal violet at room temperature for 30 minutes. The colonies were photographed, and their numbers were quantified using ImageJ software.

### Wound healing assay for cell migration

Cells were seeded in a 6-well plate, and the migration assay was performed when cells reached approximately 90% confluency. After treatment with serum-free medium for 24 hours, a scratch was created in each well using a sterile 200 μL pipette tip. The wound closure was photographed with a microscope camera every 24 hours. The wound area at 0, 24, and 48 hours was calculated using ImageJ, and the cell migration rate was determined by subtracting the wound area from the original area.

Migration rate = 1 - Wound area after migration/Wound area at 0 hours

### Screening of compounds sensitive to high LPRS HNSCC

The data processing and analysis process are as follows:

1. The gene expression matrix after drug treatment and drug sensitivity values (AUC) were obtained from the Cancer Therapeutics Response Portal (CTRP v.2.0, https://portals.broadinstitute.org/ctrp) and PRISM Repurposing dataset (19Q4, https://depmap.org/portal/prism/). Cell lines with more than 20% missing data (NA values) or derived from hematopoietic and lymphoid tissues were excluded. A total of 437 compounds were selected from the CTRP database and 1438 compounds from the PRISM database for further analysis.
2. The “calcPhenotype” function from the “oncopredict” package was used to predict drug sensitivity for HNSCC samples in this study, generating AUC values.
3. The Wilcoxon rank-sum test was used to assess differences in drug sensitivity between the high LPRS subgroup (top 20%) and the low LPRS subgroup (bottom 20%). Compounds with higher sensitivity (lower AUC values) in the high LPRS subgroup were identified using the following criteria: log2FoldChange < -0.02 (CTRP) and log2FoldChange < -0.06 (PRISM).
4. Spearman correlation analysis was performed to assess the correlation between drug sensitivity values and LPRS scores to identify positively correlated compounds. The screening criteria were set as R < -0.2 (CTRP) and R < -0.35 (PRISM).
5. Compounds identified in steps (3) and (4) were cross-referenced to extract a set of highly sensitive compounds from the CTRP and PRISM databases. In addition, the chemical structures of small molecule drugs were obtained from the PubChem chemical database (https://pubchem.ncbi.nlm.nih.gov/).

### Molecular docking analysis

The major protein structure of the key target NT5E was downloaded from the Protein Data Bank (http://www.rcsb.org, PDB). Molecular docking of key targets with small molecule drugs was performed using AutoDock Tools software (version 1.5.7). Water molecules and small molecule ligands were removed using PyMol software (http://www.pymol.org, PyMOL Molecular Graphics System). The binding activity was evaluated based on docking energy values, and the docking results were visualized.

### Cytotoxicity assay

Cell proliferation was assessed using the Cell Counting Kit-8 (MeilunBio). Cells were seeded at a density of 3000 cells per well in a 96-well plate and incubated overnight at 37°C. Cells were then co-cultured with different concentrations of drugs for 24 hours at concentrations of 0.001, 0.01, 0.1, 0.5, 1.0, 5.0, 10.0, 25.0, 50.0, 100, and 1000 μM. After discarding the supernatant, 10 μL of CCK-8 and 90 μL of culture medium were added to each well, and the cells were incubated in the dark for 2 hours. Absorbance was measured at 450 nm using a microplate reader (Model 550; Bio-Rad). Cell viability was calculated by normalizing the average OD value to the negative control.

### Statistical analysis

All statistical analyses were performed using R v4.3.1 (https://www.r-project.org/). Differences between two groups were assessed using the Wilcoxon test, while the Kruskal–Wallis test was applied for comparisons across multiple groups. Correlation analyses were conducted using Pearson’s or Spearman’s correlation coefficients, depending on data characteristics. Survival curves were generated with the Kaplan–Meier method, and OS differences were evaluated via the log-rank test. The accuracy of the LPRS in predicting OS in HNSCC patients was assessed using ROC curve analysis. A p-value of less than 0.05 was considered statistically significant. For a comprehensive description of additional experimental procedures, please refer to the **Supplementary Material section**.

## Results

### Development and characterization of LLPS-related subtypes in HNSCC patients from the TCGA cohort

**Figure 2:**
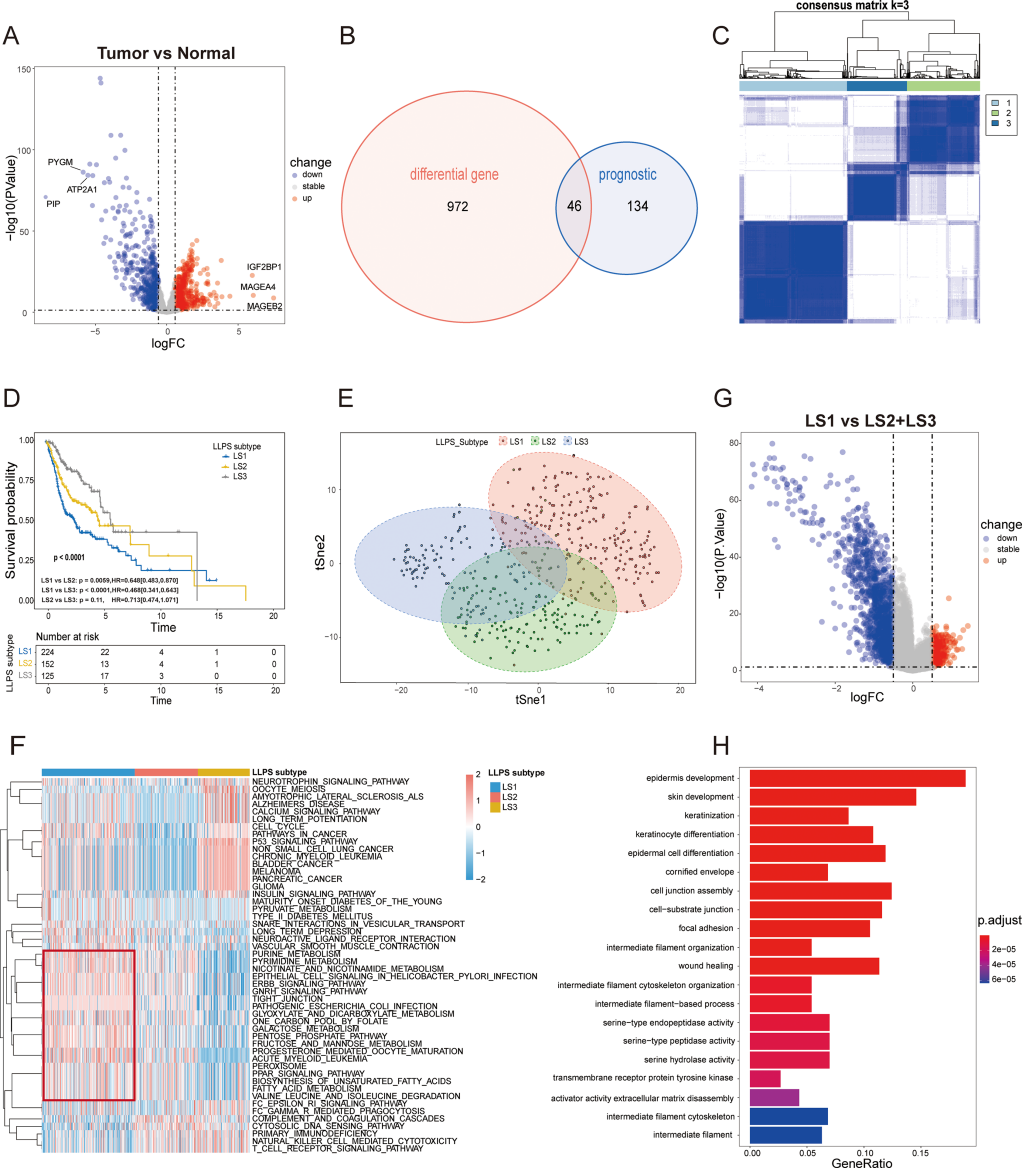
Identification of LLPS subtypes of HSNC using consensus clustering algorithm. **(A)** The volcano plot illustrates the differential expression of genes (DEGs) between HNSC tumor samples in the TCGA cohort and normal samples in GTEx cohort ( P < 0.05 and |log_2_FC|> 0.5). The top three and bottom three genes, based on log_2_FC values, are highlighted. **(B)** The Venn diagram identifies 46 prognostic DEGs associated with LLPS. **(C)** The heatmap for the result of consensus clustering. **(D)** Kaplan-Meier survival analysis reveals significant differences in overall survival (OS) among the distinct LLPS subtypes. **(E)** tSNE mapping of the expression profiles of 46 LLPS-related prognostic DEGs, effectively differentiating the LLPS subtypes. **(F)** The heatmap displays the ssGSEA Z-scores of KEGG pathways across LLPS subtypes, with blue indicating high scores and red indicating low scores. Significant differences are highlighted by the red box. **(G)** The volcano plots depict DEGs (|log_2_FC|>0.5, P<0.05) between the LS1 subtype and the combined LS2 and LS3 subtypes. **(H)** Gene Ontology (GO) enrichment analysis for the significantly upregulated genes in the LS1 subtype.

We sourced detailed information on 3,541 human LLPS-related genes from the DrLLPS database (Additional file: **Supplementary Table S2**). Transcriptomic data for these genes were sourced from the TCGA and GTEx databases. A heatmap in **Supplementary Figure S1A** illustrates the expression differences of LLPS-related genes between HNSCC tumors and normal tissues. Differential analysis identified 1,018 LLPS-related differentially expressed genes (DEGs), with 524 upregulated and 494 downregulated in HNSCC samples compared to normal tissues (**Figure 2A**; Additional file: **Supplementary Table S3**). A total of 46 prognostic LLPS-related DEGs were identified by intersecting these DEGs with those derived from univariate Cox regression analysis (Additional file: **Supplementary Table S4**; **Figure 2B**). The top 10 significantly enriched KEGG pathways for these DEGs are shown in **Supplementary Figure S1B** (Human T-cell leukemia virus 1 infection, AMPK signaling pathway, Nicotine addiction, Bladder cancer, Glycolysis/Gluconeogenesis, Amphetamine addiction, Biosynthesis of amino acids, p53 signaling pathway, Synaptic vesicle cycle, Neuroactive ligand-receptor interaction).Consensus clustering using the “ConsensusClusterPlus” R package identified k = 3 as the optimal number of subtypes, categorizing the samples into three distinct LLPS subtypes: LLPS subtype 1 (LS1), LLPS subtype 2 (LS2), and LLPS subtype 3 (LS3) (**Figure 2C**; **Supplementary Figures S1C, D**). Kaplan-Meier survival curve analysis demonstrated significant prognostic differences across these subtypes (p<0.0001), with LS1 showing the poorest survival outcomes, while LS2 and LS3 exhibited more favorable prognoses (**Figure 2D**). The accuracy of this clustering was validated using tSNE, which highlighted distinct distribution patterns among the three LLPS subtypes (**Figure 2E**). The heatmap in **Supplementary Figure S1E** reveals notable differences in the expression levels of 46 prognostic LLPS-related genes. To delve deeper into the molecular mechanisms linked to the LLPS subtypes in HNSCC, we conducted ssGSEA on transcriptome data using the KEGG dataset from the MSigDB database. The results, quantified by ssGSEA Z-scores, are displayed in heatmaps (**Figure 2F**). The unfavorable prognosis of LS1 may be associated with pathways like ERBB signaling, fatty acid metabolism, unsaturated fatty acid biosynthesis, the pentose phosphate pathway, fructose and mannose metabolism, and tight junctions, all of which likely play roles in promoting cancer cell proliferation, survival, migration, invasion, and metastasis. To corroborate these findings, 205 upregulated genes in LS1 were identified (|log_2_FC|>0.5, p<0.05) (**Figure 2G**), and subsequent GO enrichment analysis showed that the functional profiles of these genes were consistent with the ssGSEA results (**Figure 2H**), offering some explanation for the low survival rate observed in LS1.

### In-depth analysis of genomic alterations across LLPS-related subtypes

**Figure 3:**
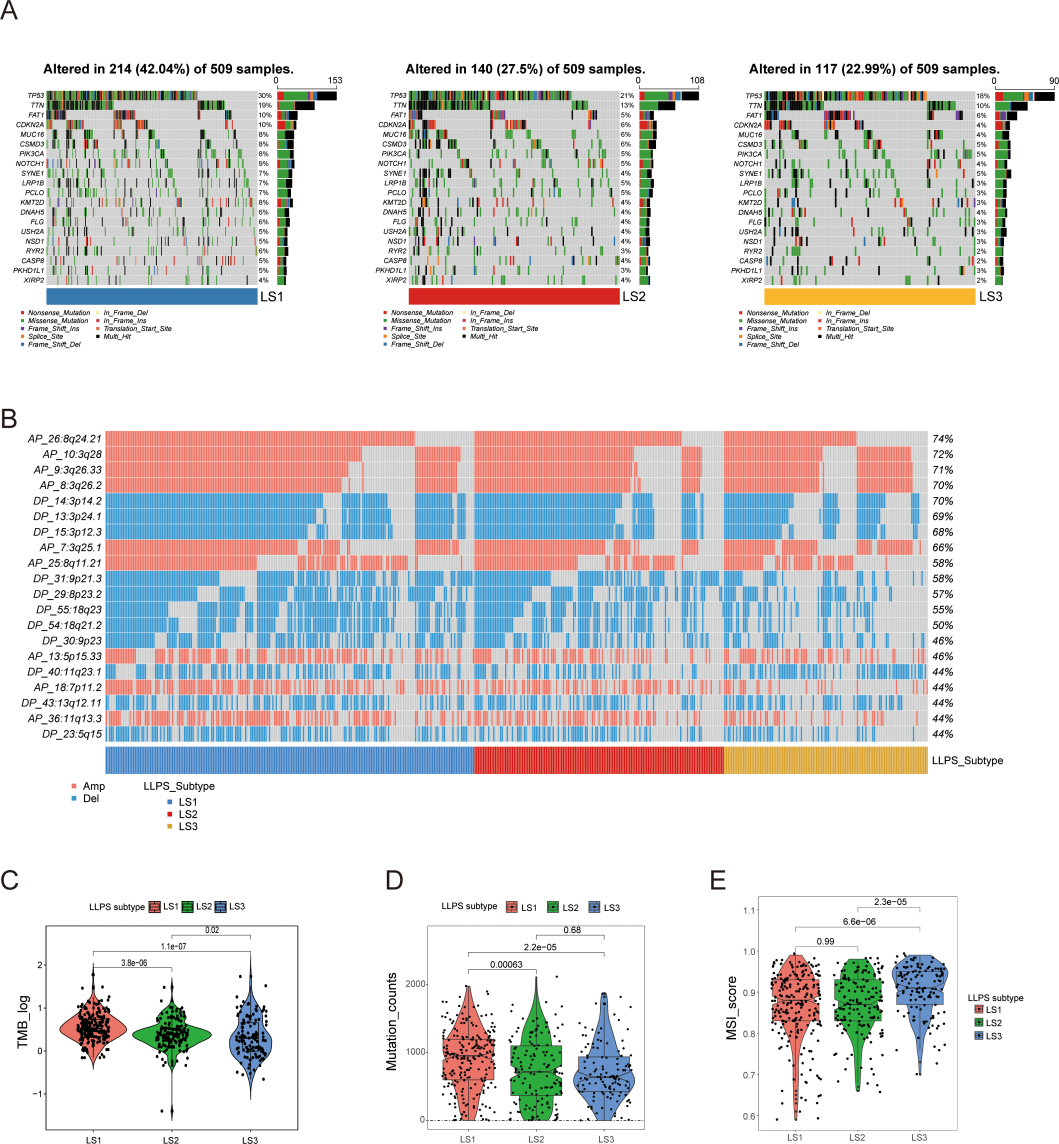
Comprehensive analysis of genomic alterations among LLPS subtypes. **(A)** Comparison of somatic mutations among LLPS subtypes. **(B)** Comparison of copy number variation (CNV) profiles among LLPS subtypes. **(C, D)** Comparison of TMB and mutation counts among LLPS subtypes. **(E)** MSI score comparison across LLPS subtypes.

To understand the genomic differences among LLPS subtypes, we analyzed somatic mutation and CNA profiles. The mutation spectrum analysis indicated that LS1 exhibited a higher frequency of TP53 mutations compared to the other subtypes (**Figure 3A**). TP53, the most commonly mutated gene in HNSCC, is associated with reduced immune activity and poor prognosis (24). **Figure 3B** illustrates the CNA characteristics among LLPS subtypes. Compared to LS2 and LS3, the LS1 subtype exhibits higher genomic instability, which may be associated with increased tumor malignancy, proliferative capacity, and resistance to therapy. LS1 also showed significantly higher levels of TMB and mutations, which were notably greater than those observed in LS2 and LS3 (**Figurse 3C, D**). Additionally, LS3 exhibited a higher microsatellite instability (MSI) score than both LS1 and LS2 (**Figure 3E**), suggesting that LS3 patients may have a greater likelihood of benefiting from ICI therapy. In conclusion, these findings not only explain the poor prognosis associated with LS1 but also suggest that LS3 may be more responsive to ICI treatment.

### TIME and immunotherapeutic responses across different LLPS-related subtypes

**Figure 4:**
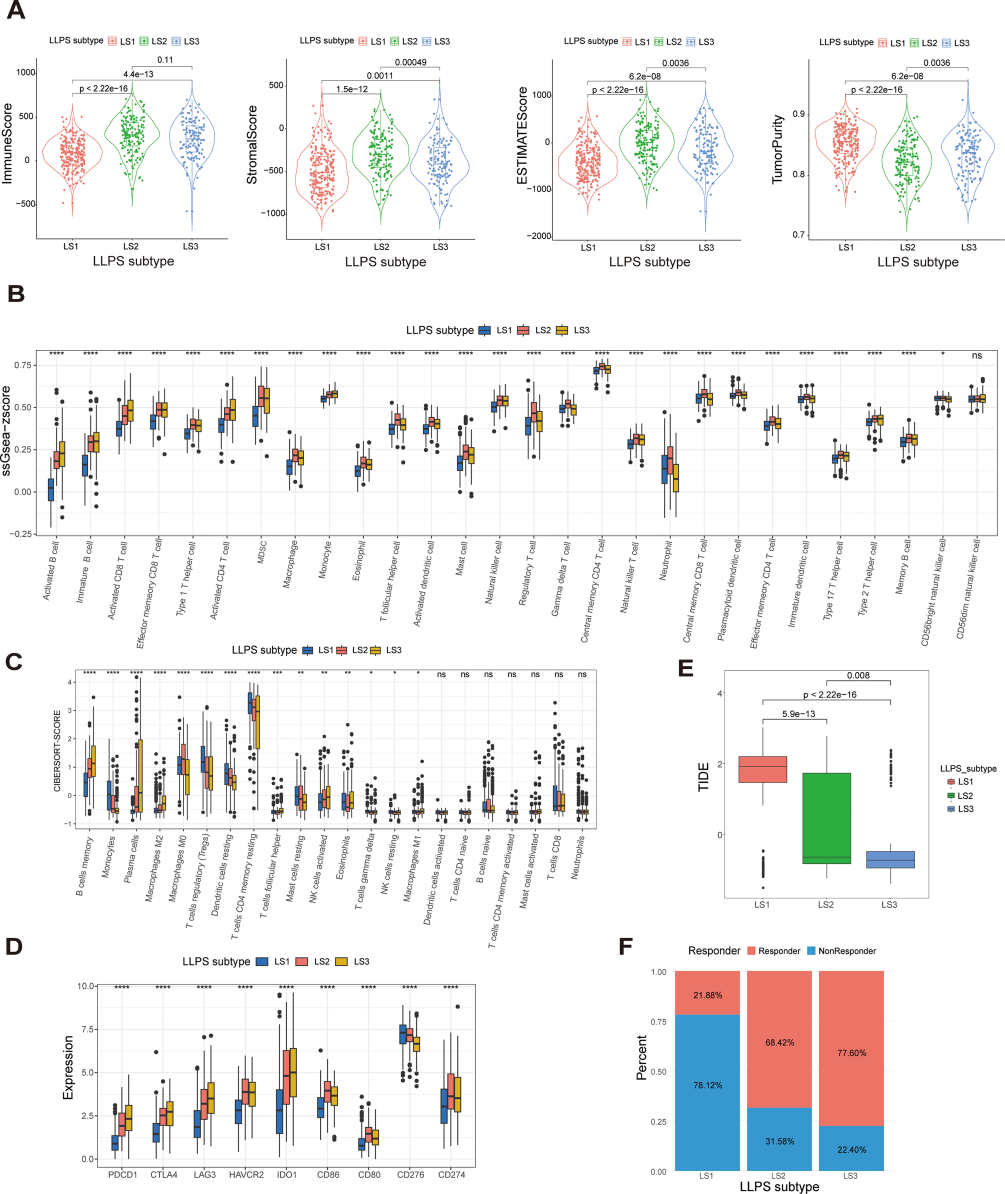
Different TIME patterns and immunotherapeutic responses of LLPS subtypes. **(A)** Violinplot of immune scores, stromal scores, ESTIMATE scores, and tumor purity across LLPS subtypes. **(B)** Immune cell infiltration levels and immune functions quantified by ssGSEA Z-scores among LLPS subtypes. **(C)** Comparison of the ratios of 22 immune cell types quantified among LLPS subtypes using the CIBESORT algorithm. **(D)** Comparison of immune checkpoint expression levels among LLPS subtypes. **(E)** TIDE score comparison across LLPS subtypes. **(F)** Proportion of ICI therapy responders predicted by the TIDE algorithm among LLPS subtypes. *:p<0.05; **:p<0.01; ***:p<0.001; ****:p<0.0001; ns, No statistical significance.

Growing evidence suggests that LLPS may play key roles in regulating the tumor immune microenvironment (TIME) and influencing immunotherapy sensitivity (16). Using the ESTIMATE algorithm, we assessed the tumor microenvironment in HNSCC samples, finding that the LS1subtype had significantly lower immune, stromal, and ESTIMATE scores, along with higher tumor purity, in contrast to LS2 and LS3, which showed the opposite trends (**Figure 4A**).

Subsequently, we utilized the immune score to classify patients with HNSCCs into high- and low-immunity subtypes. Notably, LS1 mainly consisted of hypoimmune subtypes, whereas LS2 and LS3 were characterized by hyperimmune subtypes, suggesting stronger immune functionality in the latter two (**Supplementary Figure S2**). The distribution of ssGSEA Z-scores for 28 immune signatures is illustrated in **Figure 4B**, with LS2 and LS3 showing higher immune cell infiltration.

Additionally, we used the CIBERSORT algorithm to examine the distribution of the 22 immune cell types across LLPS subtypes, revealing distinct immune cell compositions (**Figure 4C**). We also assessed the expression of mRNAs encoding key immune checkpoint proteins among these subtypes, finding that LS2 and LS3 exhibited significantly higher levels of several of them, including *PDCD1*, *CTLA4* and its ligands (*CD80* and *CD86*), *LAG3*, *HAVCR2*, *IDO1*, and *CD274*, compared to LS1 (**Figure 4D**).

Since LS1 exhibits lower immune activity, while LS2 and LS3 show stronger immune cell infiltration and higher levels of immune checkpoints, we classified LS2 and LS3 as “hot tumors” and LS1 as a “cold tumor”. Given the advantages of ICI therapy, we employed the TIDE algorithm to predict the responsiveness of different LLPS subtypes to ICI treatment. LS3 exhibited a significantly lower TIDE score compared to LS1 and LS2, indicating a higher probability of responding to ICI therapy (**Figure 4E**). Additionally, the proportion of responders in LS3 was nearly four times higher than in LS1 (**Figure 4F**). These insights underscore the significant impact of LLPS patterns on regulating the TIME and shaping responses to immunotherapy in HNSCCs.

### Construction of the LPRS and verification of its prognostic value

**Figure 5:**
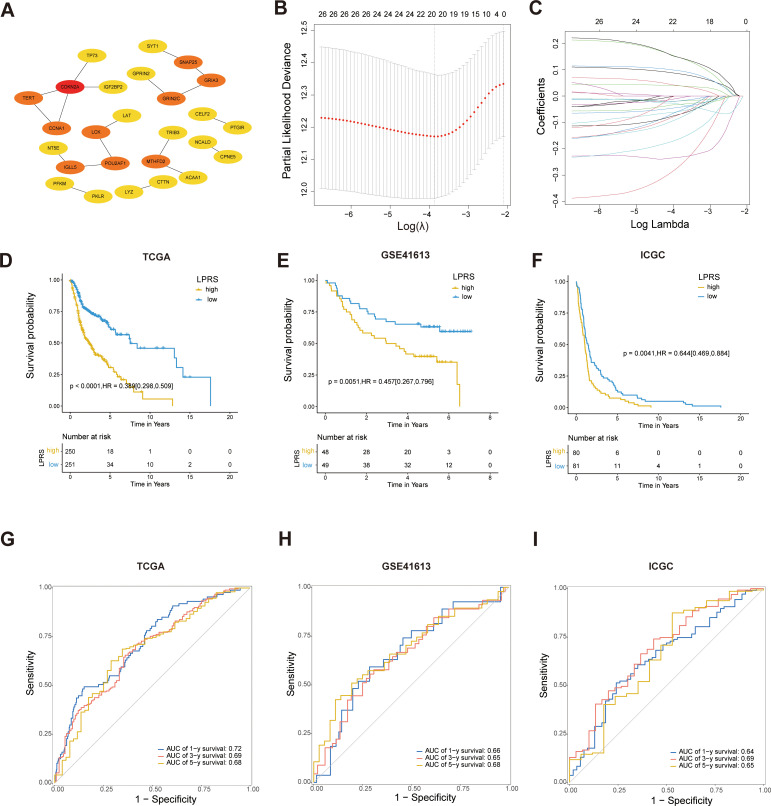
Construction and validation of prognostic models based on LLPS. **(A)** PPI network screening to identify hub genes. **(B, C)** Identification of twenty prognostic LLPS-related genes through LASSO regression and 10-fold cross-validation. **(D–F)** Kaplan-Meier survival curves for LPRS in the TCGA, GSE41613, and ICGC cohorts. **(G–I)** ROC curves for LPRS in the TCGA, GSE41613, and ICGC cohorts to validate the accuracy of the prognostic model.

To identify LLPS-related hub genes, we analyzed 46 LLPS-prognosis-related DEGs using the STRING database to explore their PPIs. Employing cytoHubba analysis, 26 hub genes were identified (**Figure 5A**).

Next, LASSO regression analysis identified 20 significant genes (*LAT*, *TERT*, *TRIB3*, *CDKN2A*, *ACAA1*, *CCNA1*, *TP73*, *CTTN*, *PKLR*, *MTHFD2*, *GPRIN2*, *CELF2*, *LCK*, *SNAP25*, *SYT1*, *NCALD*, *CPNE5*, *GRIA3*, *NT5E*, and *PFKM*) that were used to develop the LPRS (**Figures 5B, C**). The LASSO coefficients for each gene within this signature were meticulously detailed, designating 13 genes as protective and 7 as risk factors influencing survival outcomes (**Supplementary Figure S3A**). The Kaplan-Meier survival curves for these genes further substantiated their roles (**Supplementary Figure S3B**). To explore the functional connections and correlations between these genes, we performed GO enrichment analysis and correlation analysis. The genes showed a high degree of correlation with each other (**Supplementary Figure S3C**).Additionally, the pathways enriched by these genes suggest that they may be associated with cell proliferation and LLPS (**Supplementary Figure S3D**), such as the Purine Nucleoside Diphosphate Metabolic Process, Ribonucleoside Diphosphate Metabolic Process, Synaptic Vesicle Fusion to Presynaptic Active Zone Membrane, and Vesicle Fusion to Plasma Membrane.

Subsequently, the LPRS for each patient with HNSCC was computed by aggregating the expression levels of each gene, weighted by their corresponding LASSO coefficients. The patients were stratified into high- and low-LPRS groups based on the median LPRS score. Kaplan-Meier survival analysis revealed that patients in the high-LPRS group exhibited significantly worse overall survival in TCGA dataset (**Figure 5D**). The robustness of this prognostic model was confirmed through validation with two additional datasets, GSE41613 and ICGC-HNSC, where a high LPRS consistently correlated with poor survival (**Figures 5E–F**).

Furthermore, to assess the predictive accuracy of the LPRS for overall survival, ROC curves for 1-, 3-, and 5-year survival predictions were constructed, demonstrating AUC values exceeding 0.68, which underscored the potential utility of the LPRS as a reliable prognostic tool in clinical settings (**Figure 5G**). The high predictive accuracy of the LPRS was further validated in the external datasets GSE41613 and ICGC-HNSC (**Figures 5H–I**), reinforcing its efficacy in clinical prognosis.

### Association of LPRS with clinicopathological features, TIME patterns, and its predictive role in ICI therapy response

**Figure 6:**
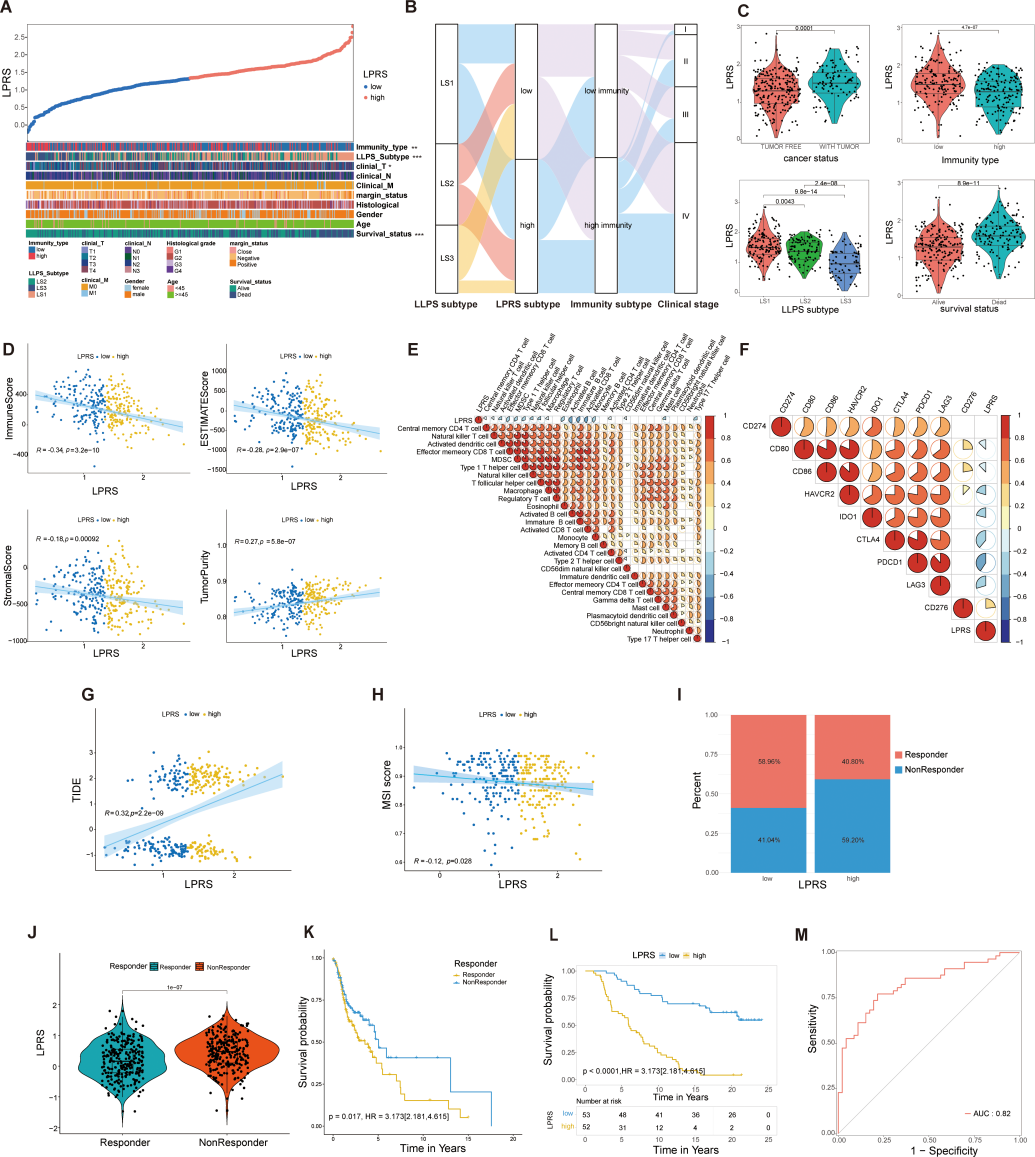
Correlation of LPRS with clinicopathological features and TIME pattern in the TCGA cohort and its role in predicting response to ICI therapy. **(A)** Overview of the relationship between LPRS and various clinicopathological features in HNSC patients. **(B)** Alluvial diagram illustrating the transitions in LLPS subtypes, immunity subtypes, clinical stage, and LPRS. **(C)** Comparison of LPRS across cancer status, immunity type, LLPS subtype, and survival status. **(D)** Correlation analysis of LPRS with immune scores, stromal scores, ESTIMATE scores, and tumor purity. **(E)** Correlation between LPRS and the ssGSEA Z-scores of 28 immune signatures. **(F)** Correlation analysis between LPRS and immune checkpoint expression.**(G, H)** Correlation of LPRS with TIDE and MSI scores in the TCGA cohort. **(I)** The proportion of ICI therapy responders predicted by the TIDE algorithm in high-LPRS and low-LPRS subgroups within the TCGA cohort. **(J)** Comparison of LPRS levels between responders and non-responders in the TCGA cohort. **(K)** Survival analysis between responders and non-responders in the TCGA cohort. **(L, M)** Accuracy and reliability of LPRS in predicting the efficacy of PD-L1 immunotherapy in patients (IMvigor210 cohort: AUC = 0.82, Kaplan-Meier plot p<0.0001). *:p<0.05; **:p<0.01; ***:p<0.001; ****:p<0.0001; ns, No statistical significance.

To elucidate the clinical relevance of the LPRS within TCGA cohort, we ranked the LPRS from lowest to highest to analyze its association with clinicopathological features. We observed significant differences in survival status, clinical T stage, high- and low-immune subtypes, and LLPS subtype (**Figure 6A**). An alluvial plot was used to depict the transitions across LLPS and LPRS classifications, immune subtypes, and clinical stages among patients (**Figure 6B**). Most LS1 subtype patients were found in the high LPRS subtype, low immune subtype, and clinical stage IV, while the majority of LS3 patients were distributed in the low LPRS subtype, high immune subtype, and clinical stage IV. This suggests the precise predictive ability of LPRS and indicates that ICI treatment may be more effective for patients with low LPRS. Additionally, we assessed the LPRS levels across different clinicopathological subgroups, finding that significantly elevated LPRS levels were related to malignant features and mild TIME. Notably, the LPRS was higher in the low-immune subtype than in the high-immune subtype. When comparing LLPS-related subtypes, LPRS levels were highest in LS1 (**Figure 6C**).

Given the association between the LPRS and various immune subtypes, we delved deeper into the relationship between TIME patterns and LPRS. In the TCGA cohort, LPRS was inversely correlated with immune, stromal, and ESTIMATE scores and positively correlated with tumor purity, suggesting a decrease in immune and stromal cell infiltration as the LPRS increased (**Figure 6D**). The relationships between the LPRS and 28 immune signatures are depicted in a correlation heatmap (**Figure 6E**). Similarly, we performed a correlation analysis between LPRS of each LLPS subtype and 28 immune features, and the results were consistent with the previous findings (**Supplementary Figure S4A**).

Additionally, a negative correlation was observed between the LPRS and the expression levels of most immune checkpoint molecules (**Figure 6F**), underscoring the complex interactions between the LPRS and immune dynamics in HNSCC.To explore the relationship between LPRS of LLPS subtypes and immune checkpoints, we also performed a correlation analysis for each LLPS subtype, and the results were consistent with the previous findings (**Supplementary Figure S4B**). Subsequently, we performed a further correlation analysis between LPRS and the infiltration levels of 22 immune cell types using the CIBERSORT algorithm. The results revealed negative correlations with activated NK cells, resting dendritic cells, neutrophils, and activated dendritic cells, while showing positive correlations with resting CD4 memory T cells, naive B cells, and regulatory T cells (**Supplementary Figure S4C**).

To validate the predictive role of LPRS in ICI treatment response, our analysis of the TCGA cohort revealed that patients with lower LPRS scores had reduced TIDE levels and elevated MSI scores (**Figures 6G, H**). According to the TIDE algorithm, a larger fraction of ICI treatment responders were observed in the low-LPRS subgroup than in the high-LPRS subgroup (**Figure 6I**). Moreover, the LPRS values of responders were consistently significantly lower than those of non-responders, with significant survival differences observed (**Figures 6J, K**). This leads us to hypothesize that patients with lower LPRS are more likely to benefit from ICI treatment compared to those with higher LPRS.

To confirm our findings, we further assessed the predictive ability of the LPRS in an independent ICI treatment cohort (IMVigor210, an anti-PD-L1 cohort). In this cohort, a significantly higher proportion of patients in the low-LPRS subgroup achieved complete response (CR) or partial response (PR) (**Supplementary Figure S4D**). Patients exhibiting a CR displayed notably lower LPRS levels than those with stable disease (SD) or progressive disease (PD) (**Supplementary Figure S4E**). To verify the accuracy of the LPRS in predicting ICI treatment response, we also constructed KM survival curves and ROC curves for the IMVigor210 cohort (**Figures 6L, M**). We found that the survival rates of low-LPRS patients were significantly better than that of high-LPRS patients and that the AUC value of the ROC curve reached 0.82. These results indicate the high accuracy and reliability of the LPRS in predicting ICI treatment response.

Overall, these results not only demonstrate the association between LPRS and clinicopathological characteristics, as well as TIME, but also highlight the strong correlation between LPRS and ICI therapy response, suggesting its potential as a reliable predictor for clinical outcomes.

### Screening and molecular docking of potential small molecule compounds targeting high LPRS

**Figure 7:**
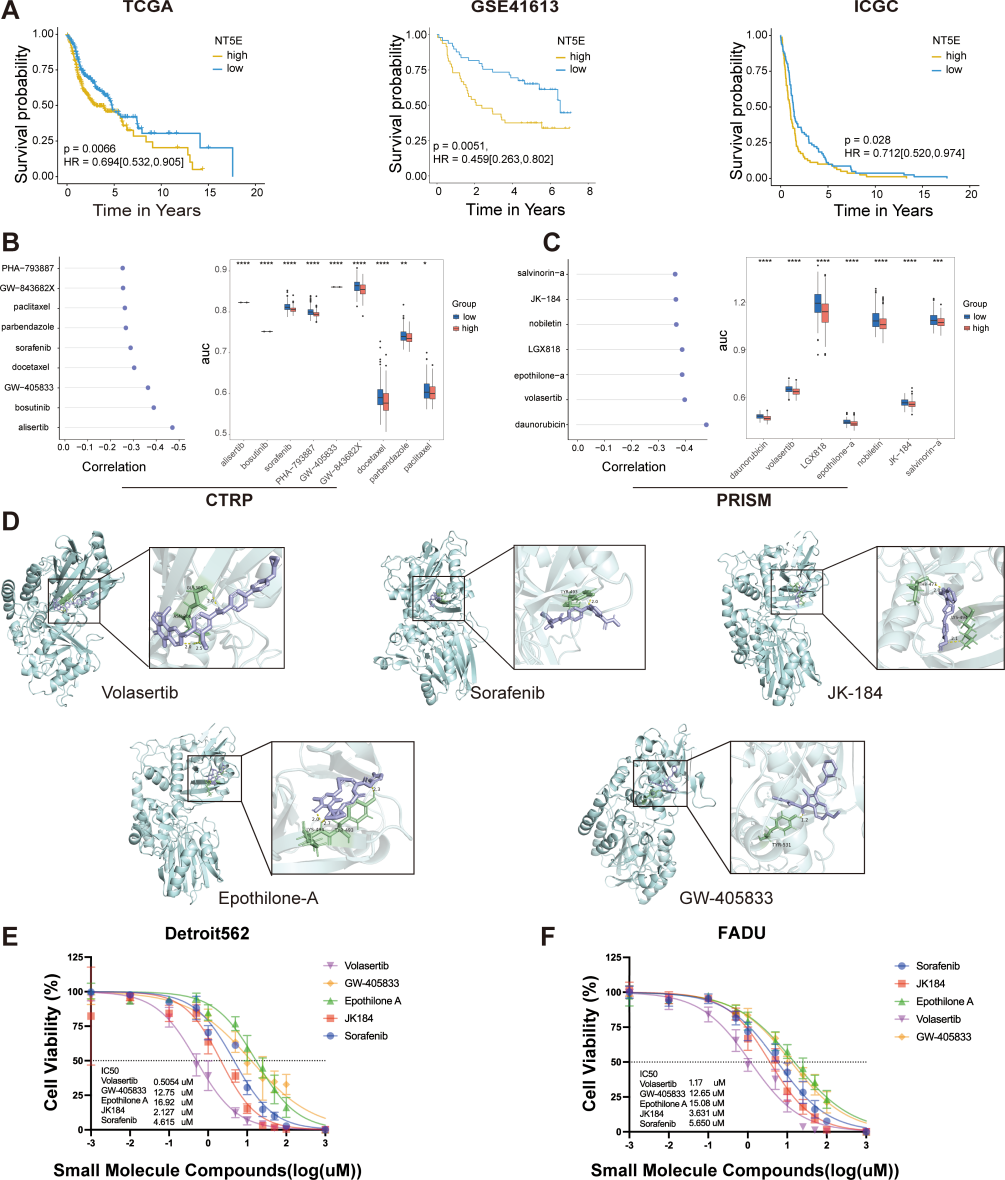
LPRS Potential Compound Screening. **(A)** Kaplan-Meier plots illustrating the prognostic value of NT5E(CD73) in the TCGA, GSE41613, and ICGC cohorts. **(B, C)** Spearman correlation analysis results and differential drug responses for potential compounds identified from the CTRP and PRISM databases. **(D)** Visualization of molecular docking results for the top five potential compounds, ranked by molecular docking scores. **(E, F)** Cytotoxicity assays for the top five potential compounds were performed in Detroit562 and FADU cell lines, including the calculation of drug IC50 values. *:p<0.05; **:p<0.01; ***:p<0.001; ****:p<0.0001; ns, No statistical significance.

Since patients with lower LPRS are more likely to benefit from ICI therapy, we aimed to identify potential small molecules that could be more beneficial for patients with high LPRS.We adopted a comprehensive strategy to identify potential small-molecule compounds for use in HNSCC patients exhibiting a high LPRS. It has been reported that NT5E (CD73) generates extracellular adenosine, mediates immune escape, and promotes tumor growth and metastasis, making it a crucial target for immunotherapy (25). Additionally, NT5E (CD73) is the only gene that shows significant differences in overall survival across multiple datasets, including TCGA, GSE41613, and ICGC (**Figures 7A**; **Supplementary Figure S5**). Therefore, we have focused on NT5E (CD73) and selected it as a key target for studying LPRS.

Drug susceptibility data were sourced from the CTRP and PRISM databases, and the “oncopredict” R package was utilized to predict drug response within the TCGA-HNSC cohort. Prior to further analysis, we confirmed the reliability of the predicted drug response data. ICAM1, a cell surface glycoprotein present on leukocytes and endothelial cells, has been implicated in recent research, which suggests that its activation enhances the stemness of HNSCC cells, potentially contributing to docetaxel resistance (26). Patients were stratified into two groups based on the median ICAM1 expression levels, revealing that the estimated AUC value of docetaxel was significantly elevated in the ICAM1_high group, aligning with previous experimental observations (**Supplementary Figure S6A**).

We identified potential compounds using the CTRP and PRISM databases. From the CTRP database, we selected nine candidate compounds: alisertib, bosutinib, sorafenib, PHA-793887, GW-405833, GW-843682X, docetaxel, parbendazole and paclitaxel. Similarly, from the PRISM database, we identified seven candidate compounds: daunorubicin, volasertib, LGX818, epothilone-A, nobiletin, JK184 and salvinorin-A (**Figures 7B, C**). The molecular structures of these small molecules are shown in **Supplementary Figure S6B**. Subsequently, we conducted molecular docking studies to investigate the interaction of these compounds with the key LPRS target NT5E (CD73). We focused on the top five compounds with the highest binding affinities to NT5E (CD73): Volasertib, Sorafenib, JK-184, Etoposide-A, and GW-405833. Their docking results were visualized (**Figure 7D**; **Supplementary Table S5**). While these compounds are well-known, none have been reported to specifically target NT5E (CD73) in HNSCC. To evaluate the therapeutic potential of these five compounds for high LPRS HNSCC patients, we performed IC50 assays. The results indicated that Volasertib exhibited the greatest therapeutic potential, making it a promising candidate for treating high LPRS HNSCC patients (**Figures 7E, F**).

### Analysis and functional validation of NT5E gene expression levels

**Figure 8:**
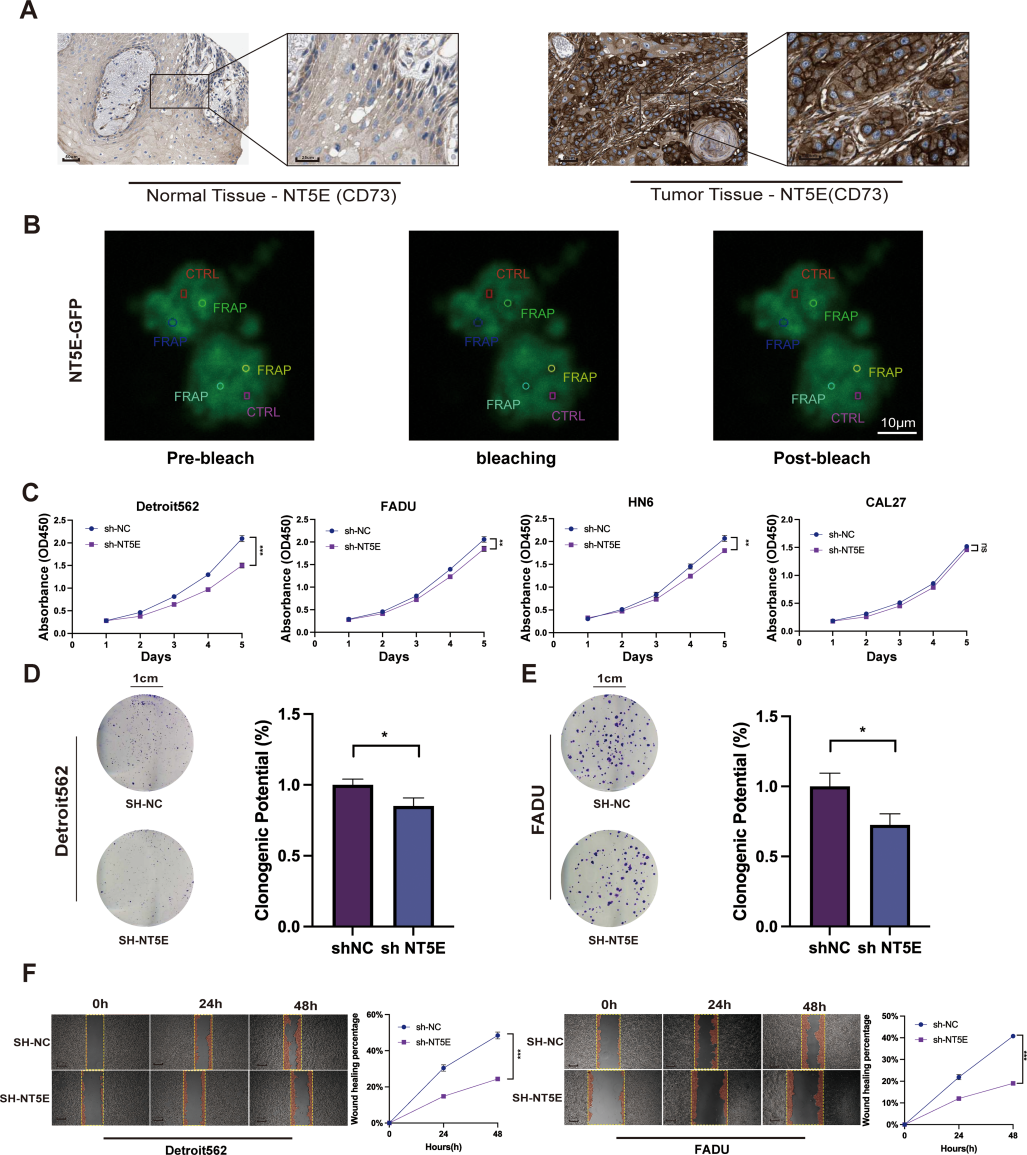
Expression and function of the NT5E (CD73) gene in LPRS. **(A)** Immunohistochemistry (IHC) images from the HPA database comparing NT5E (CD73) protein expression in normal and tumor tissues in HNSC. **(B)** Fluorescence recovery after photobleaching (FRAP) experimental images of cells overexpressing NT5E-GFP. The red rectangle represents the CTRL, and the other circles represent FRAP. **(C)** CCK8 assays evaluating the impact of NT5E (CD73) knockdown on the proliferation of HNSC cell lines. **(D, E)** Colony formation assays detecting changes in cell proliferation following NT5E (CD73) knockdown. **(F)** Scratch assays assessing the effect of NT5E(CD73) knockdown on cell migration. *:p<0.05.

To validate the reliability of LPRS, we performed expression analysis and *in vitro* functional assays on NT5E (CD73), the key target gene of LPRS. Results indicated that NT5E (CD73) mRNA expression was notably higher in HNSCC tumor tissues compared to normal tissues (**Supplementary Figure S7A**). To assess NT5E (CD73) expression at the protein level, immunohistochemistry images from the HPA database were utilized. NT5E (CD73) protein expression in HNSCC was markedly higher than that in normal tissues (**Figure 8A**). Additionally, we explored the correlation between NT5E (CD73) protein expression and mRNA expression. Using data from the TCGA database, we demonstrated the relationship between NT5E (CD73) protein expression and mRNA expression, which helps to clarify the link between gene and protein expression levels. The results showed a positive correlation between NT5E (CD73) protein expression and mRNA expression (**Supplementary Figure S7B**).

We validated the knockdown of NT5E (CD73) using RT-qPCR, followed by further functional assays (**Supplementary Figure S7C**). To investigate the potential involvement of NT5E(CD73) in LLPS formation, we analyzed the dynamic assembly and rapid exchange properties of NT5E (CD73) induced liquid condensates by measuring the fluorescence recovery after photobleaching (FRAP). After photobleaching, the NT5E-GFP puncta recovered fluorescence within seconds (**Figure 8B**). This result indicates that NT5E (CD73) significantly influences the formation and recovery of LLPS structures, suggesting a potential functional link between NT5E (CD73) expression and LLPS.

To elucidate the role of NT5E (CD73) in HNSCC proliferation and migration, we employed CCK8 assays, which revealed that knockdown of NT5E (CD73) significantly inhibited the proliferation of Detroit562, FADU, HN6, and other cell lines compared with that of controls (**Figure 8C**). The two cell lines that exhibited the most pronounced differences after NT5E (CD73) knockdown were chosen for additional functional experiments. Colony formation assays then corroborated these findings, demonstrating that cell proliferation was markedly inhibited in the SH-NT5E lines compared to the NC group (**Figures 8D, E**).

We then divided HNSCC patients into high and low NT5E (CD73) expression groups and performed GSEA enrichment analysis. The results indicated that NT5E (CD73) is not only associated with cell proliferation but also closely linked to cell migration, such as in pathways like FOCAL_ADHESION and ECM_RECEPTOR_INTERACTION (**Supplementary Figure S7D**). Therefore, we conducted a wound healing assay to assess the effect of NT5E knockdown on HNSCC migration (**Figure 8F**). Compared to the NC group, the migration rate of the NT5E (CD73) knockdown cell lines was significantly reduced.

## Discussion

Increasing evidence underscores the role of LLPS in both tumorigenesis and cancer progression (27). Furthermore, research has demonstrated that LLPS contributes to the formation of various TIME patterns and influences immune signaling regulation (28, 29). Thus, a thorough investigation of LLPS-related biomarkers is crucial for uncovering new tumor subtypes, making accurate prognoses and predicting responses to immunotherapy.

The response to ICI therapy is also a priority. This therapy has led to significant advancements in cancer treatment, but there is substantial heterogeneity in treatment response. Therefore, our study focused on the predictive capacity of the LPRS in determining responses to ICI therapy.

We attempted to establish prognostic signatures by analyzing LLPS-related genes to be able to predict which patients are sensitive to ICI treatment based on typing in order to make accurate HNSCC prognoses and guide personalized treatments. In this study, we concentrated on HNSCC patients and used the expression profiles of 46 prognostic LLPS-related DEGs to classify 501 HNSCC patients into three distinct LLPS subtypes through a consensus clustering algorithm. We observed marked differences between these subtypes in terms of prognosis, functional enrichment, genomic alterations, TIME patterns, and immunotherapy responses. To facilitate personalized and comprehensive assessments, we constructed a prognostic signature known as the LPRS using PPI networks and LASSO regression. To verify the predictive ability of the LPRS for ICI treatment response, we validated it using an IMVigor-independent cohort. Our findings indicated that the LPRS correlates strongly with LLPS subtype, clinical features, and TIME patterns in HNSCC patients, thus demonstrating exceptional predictive capabilities for ICI treatment responsiveness.

Tumors acquire hallmark characteristics, such as sustained proliferation, angiogenesis, EMT, and genomic rearrangements, through various mechanisms. However, the field of LLPS has changed our understanding of how tumors develop these malignant traits (27). For instance, estrogen can induce MYC to form condensates through an LLPS-mediated mechanism, thereby enhancing VEGF expression and promoting angiogenesis (30). Additionally, LLPS involving transcriptional coactivators, such as YAP/TAZ, plays a crucial role in EMT and cancer aggressiveness (31, 32). Abnormal LLPS of the ENL protein gathers at specific genomic loci, recruits numerous transcription complexes, and potentially leads to genomic rearrangements characteristic of cancer (33, 34). In our analysis, different LLPS subtypes in HNSCC demonstrated distinct behaviors in terms of the KEGG pathways involved. Notably, the LS1 subtype exhibited increased activity in pathways such as erbB signaling, fatty acid metabolism, biosynthesis of unsaturated fatty acids, the pentose phosphate pathway, fructose and mannose metabolism, and tight junctions. These pathways likely contribute to an increased capacity for tumor proliferation, survival, migration, invasion and metastasis (35, 36). These findings may explain the poor prognosis of LS1.

According to traditional classification, tumors can be categorized into three distinct TIME phenotypes: immune-inflamed, immune-excluded and immune-desert. Historically, HNSCC have predominantly been associated with an immune-desert phenotype, suggesting that these tumors are more adept at evading immune system attacks, complicating treatment. Recent advances in understanding the role of LLPS in both innate and adaptive immunity have offered new perspectives on this process. For example, the substitution of GMP-AMP synthase with LLPS enhances the production of cyclic GMP-AMP second messengers, thereby amplifying innate immune signaling (24). Furthermore, the aggregation of biomolecules in T cell transmembrane signal receptors into clusters via LLPS may enhance signal transduction and modulate the immune response of the tumor (37). In this study, the LS2 and LS3 subtypes exhibited higher immune and stromal scores but lower tumor purity, indicating a higher presence of non-tumor components compared with other subtypes. Additionally, LS2 and LS3 showed significant immune cell infiltration, suggesting that these subtypes align with an immunoinflammatory phenotype and are likely to respond favorably to immunotherapy. Analysis using the TIDE algorithm confirmed this hypothesis, revealing that LS3 had a lower TIDE score and a higher MSI score compared to LS1 and LS2. These results suggest that the identified LLPS subtypes are useful for recognizing distinct TIME patterns and for identifying patients who may respond favorably to ICI therapy.Future studies are necessary to elucidate the precise mechanisms by which LLPS influences the formation of these TIME patterns, the knowledge of which would enhance our ability to tailor and optimize immunotherapeutic strategies for patients with HNSCCs.

Given the diverse heterogeneities among the three LLPS subtypes, constructing a prognostic signature appears feasible for quantifying such variations and facilitating personalized, integrative assessments. As anticipated, the LPRS demonstrated a strong correlation with the clinicopathological features and TIME patterns of patients with HNSCC. Furthermore, it showed significant predictive power for the prognosis and response to ICI therapy. The LPRS consists of twenty selected LLPS-related genes: *LAT*, *TERT*, *TRIB3*, *CDKN2A*, *ACAA1*, *CCNA1*, *TP73*, *CTTN*, *PKLR*, *MTHFD2*, *GPRIN2*, *CELF2*, *LCK*, *SNAP25*, *SYT1*, *NCALD*, *CPNE5*, *GRIA3*, *NT5E*, and *PFKM*, which includes one scaffold, two regulators, and seventeen clients. The scaffold LAT is a transmembrane protein essential for T cell activation, undergoing LLPS upon tyrosine phosphorylation and interacting with cytoplasmic multivalent adaptor proteins (38). The regulator TERT, when interacting with CIRP, influences TERT’s transcription and translation processes, thereby localizing the telomerase complex to the Cajal body (39). Another regulator, TRIB3, is upregulated and interacts with PML-RARα, inhibiting the assembly of PML nuclear bodies (40). Among the 17 clients identified, CDKN2A, PKLR, and MTHFD2 are involved in nucleolus formation (41, 42). TP73 and GPRIN2 participate in the assembly of PML nuclear bodies (43, 44). CELF2 is involved in stress granule formation, while SNAP25, SYT1, NCALD, CPNE5, GRIA3, NT5E, and PFKM contribute to the formation of postsynaptic densities (45). The other four clients are involved in the formation of various biomolecular condensates: ACAA1 (nucleolus, postsynaptic density); CCNA1 (centrosome, spindle pole body); CTTN (centrosome, spindle pole body, stress granule); LCK (centrosome, spindle pole body) (45–49). Currently, our understanding of these LLPS-related genes primarily focuses on their roles in biomolecular condensate formation. Future research should further investigate how LLPS processes contribute to tumor development and progression, providing deeper insights into the complex mechanisms underlying cancer biology.

Exploring individualized treatment strategies for different LPRS subgroups is of great significance for maximizing treatment effects. In addition to providing prognostic information and predicting immunotherapy responses, the LPRS can be used in precision oncology as a potential biomarker for HNSCC treatment. Although only a small number of patients benefit from targeted strategies and immunotherapy, docetaxel remains the first-line treatment for HNSCC. However, DTX resistance is common and threatens the long-term survival of patients (26). Therefore, after comprehensive screening and molecular docking, we screened out five potential compounds (volasertib, sorafenib, JK-184, epothilone-A, and GW-405833). Volasertib is a key Plk1 inhibitor with broad anticancer activity. Preclinical studies have shown strong efficacy across various cancer cell lines and tumor regression in multiple xenograft models (50). Sorafenib is a serine-threonine protein kinase inhibitor targeting bRaf, C-Raf, VEGFR, and platelet-derived growth factor receptors. When combined with radiochemotherapy, it enhances antitumor effects by inhibiting cell proliferation, colony formation, migration, and invasion (51). JK184, an anilinopyrimidine derivative, specifically inhibits Gli in the Hedgehog (Hh) pathway, showing significant promise in cancer therapy (52). Epothilone-A, a microtubule-stabilizing agent similar to paclitaxel, inhibits cell division by stabilizing microtubules and is widely used in chemotherapy (53). GW-405833 is a selective CB2 receptor agonist that has been shown to induce autophagy in pancreatic cancer cells, thereby inhibiting their growth (54). Of them, volasertib, a small molecule PLK1 inhibitor, showed the greatest therapeutic potential. Studies have shown that PLK1 expression is negatively correlated with the survival rates for HNSCC and other solid tumors (55). These findings highlight the critical role of the LPRS in drug screening and provide a promising option for the treatment of HNSCC.

To date, numerous HNSCC classifications have been based on traditional biomarkers or significant molecular indicators. For instance, TP53 mutation status is widely recognized as a crucial prognostic biomarker for HNSC (56). Our classification approach, which leverages LLPS subtyping, offers an advantage by revealing multidimensional heterogeneities. These factors include prognosis, functional enrichment, genomic alterations, TIME patterns, and, in particular, responses to immunotherapy, which is of considerable clinical relevance.

This study has some limitations. First, all analyses were conducted using retrospective data from public databases; employing prospective multicenter cohorts could enhance the reliability of the results. Secondly, due to the limited availability of immunotherapy cohorts with accessible transcriptional data and clinical information, our evaluation of the predictive capacity of LPRS for ICI therapy was restricted to data from urothelial cancer cohorts. Furthermore, while bioinformatics analysis provides valuable insights, it falls short of fully elucidating the underlying molecular mechanisms, highlighting the indispensable need for experimental evidence to advance our understanding. In this study, we focused on validating NT5E (CD73), the key target gene of LPRS, through experimental assays. However, the other 19 genes included in the LPRS model were not experimentally validated. Future studies should aim to validate these additional genes to strengthen the findings and explore their roles in HNSCC. Finally, we did not separate HPV(+) and HPV(-) subtypes in our analysis, despite the significant differences in tumorigenic mechanisms and immune microenvironment characteristics between these two groups. This is a limitation, and future studies could explore how LLPS and immune responses may vary across these subtypes.

## Conclusion

In conclusion, we successfully classified patients with HNSCC into three unique LLPS subtypes, each characterized by distinct prognostic outcomes, functional enrichment profiles, genomic alterations, TIME patterns, and immunotherapy responses. Additionally, we developed a prognostic signature, the LPRS, to facilitate personalized comprehensive assessments and to identify potential small-molecule compounds for targeted therapy. These findings offer promising avenues for enhancing customized prognostic predictions and optimizing immunotherapeutic strategies for patients with HNSCC. Further studies are required to validate and expand upon these results.

## Data Availability

The original contributions presented in the study are included in the article/**Supplementary Material**. Further inquiries can be directed to the corresponding authors.
