# Supplementary material for: Multi-omics analysis identifies a liquid-liquid phase separation-related subtypes in head and neck squamous cell carcinoma
Source: Front Oncol. 2025 Feb 26;15:1509810. doi: 10.3389/fonc.2025.1509810 (PMC11897011; doi:10.3389/fonc.2025.1509810)
Supplement: Supplementary file 1 [file DataSheet1.docx]

Supplementary Material

# Supplementary Figures and Tables

## Supplementary Figures


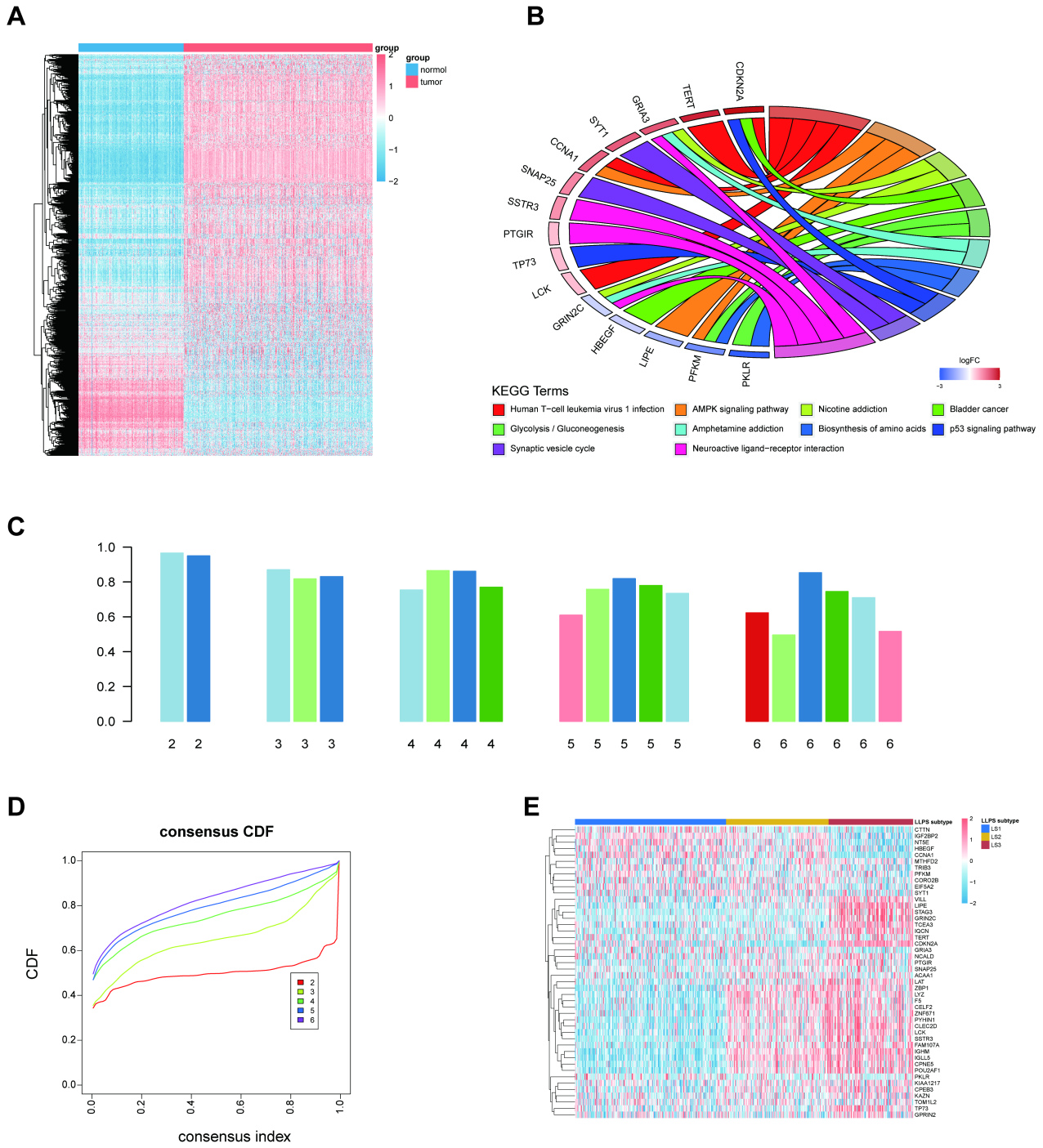


**Fig. S1** **Functional enrichment and clustering analysis of LLPS-related genes in HNSC patients. (A)** Heatmap illustrating the expression differences of LLPS-related genes between tumor samples from HNSC patients and normal tissues. **(B)** Top ten KEGG pathways enriched for the 46 prognostic differential genes. (**C-D)** Cluster-consensus value scores and CDF plots for subtype classification using consensus clustering of the 46 prognostic differential genes. (**E)** Heatmap displaying the expression levels of the 46 LLPS-related prognostic DEGs across the LLPS subtypes.


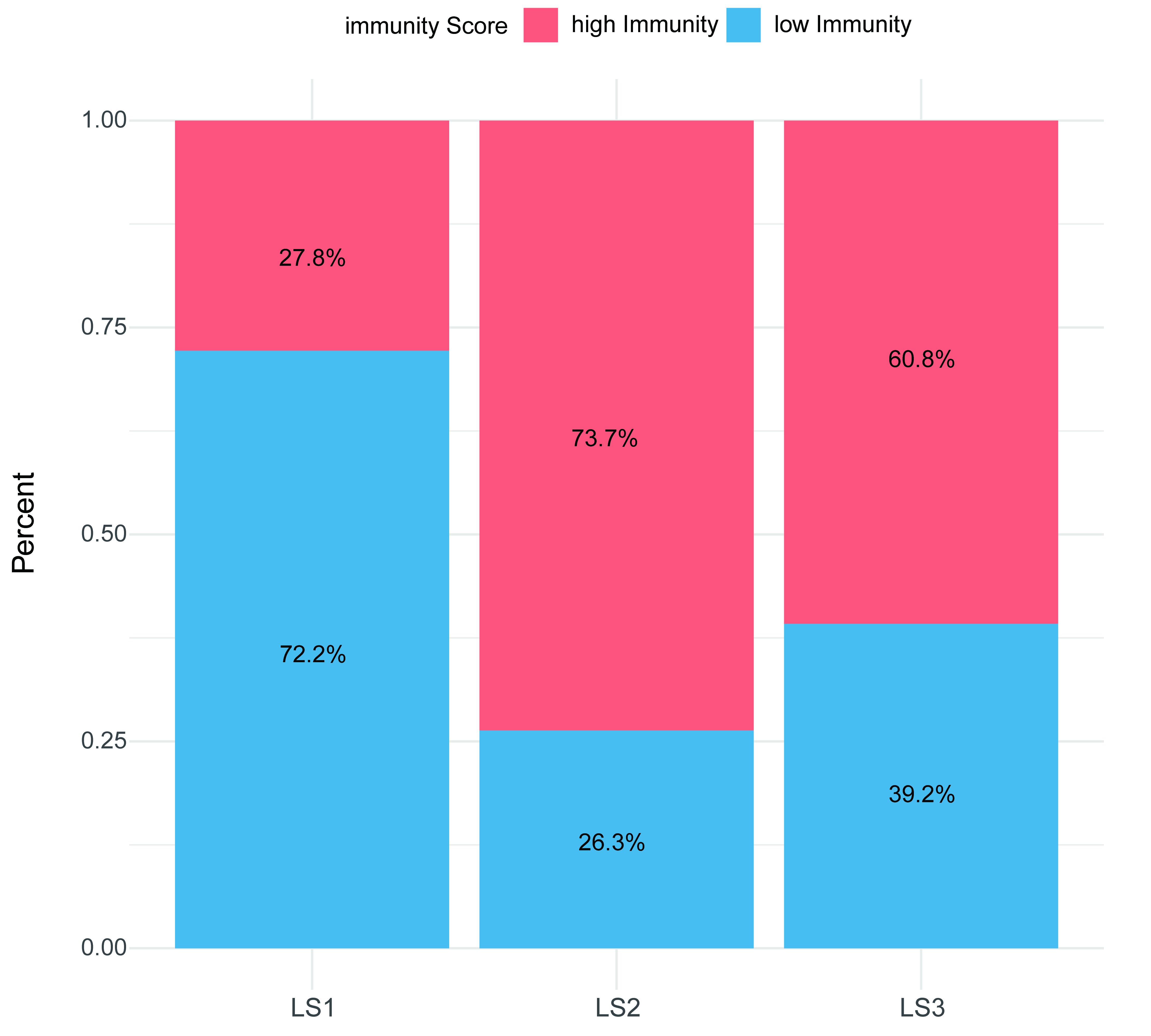


**Fig. S2 The ratio of high and low immune scores.** Bar chart showing the ratio of high and low immune scores among LLPS subtypes.


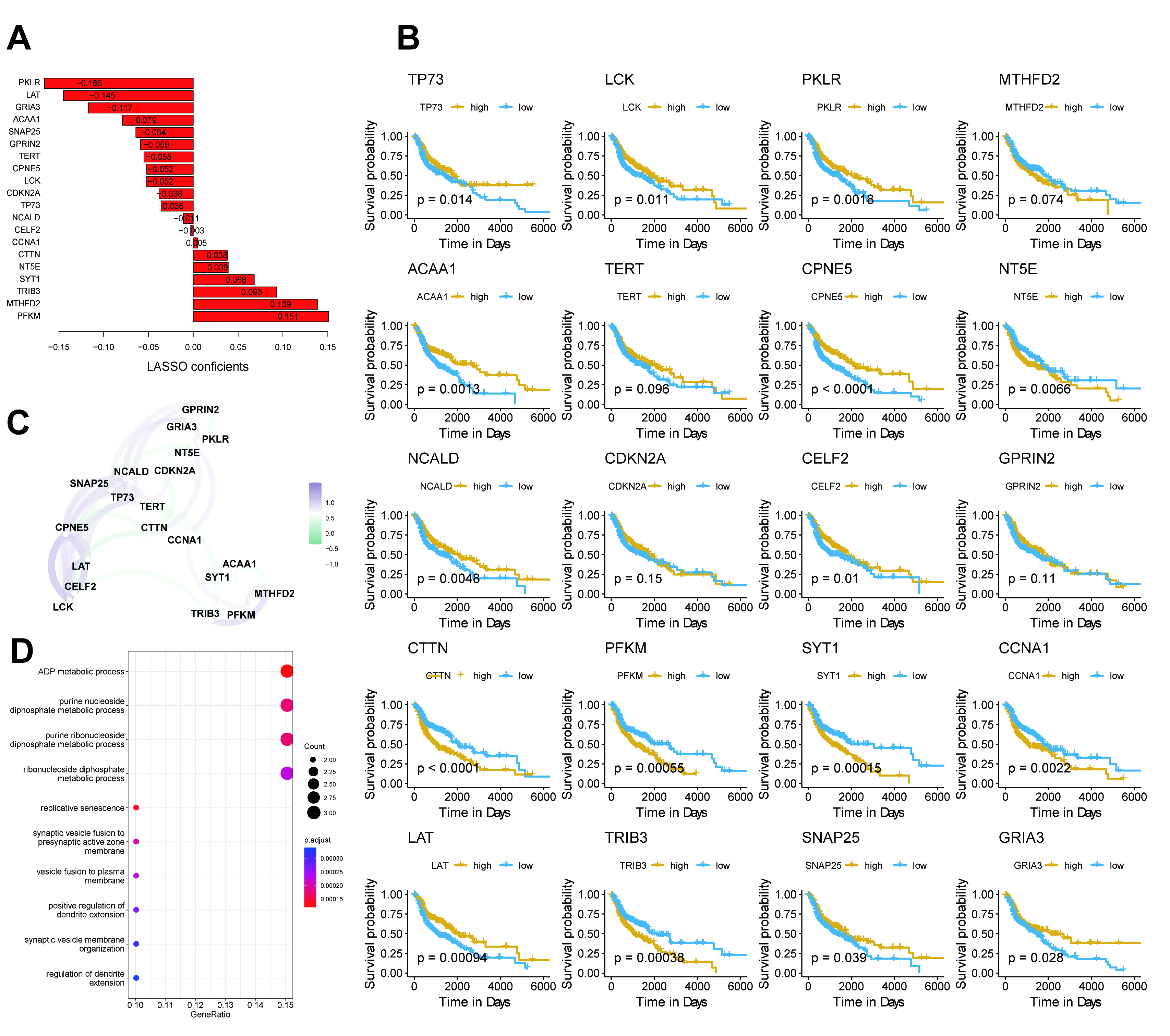


**Fig. S3 The role of the 20 hub genes screened by the model in TCGA.** **(A)** LASSO regression coefficients for the 20 hub genes. **(B)** Kaplan-Meier plot of the 20 hub genes using the LPRS median as the cutoff point. **(C)** Correlation network diagram between 20 hub genes. **（D）**GO enrichment analysis was performed on these 20 hub genes to obtain their pathways.


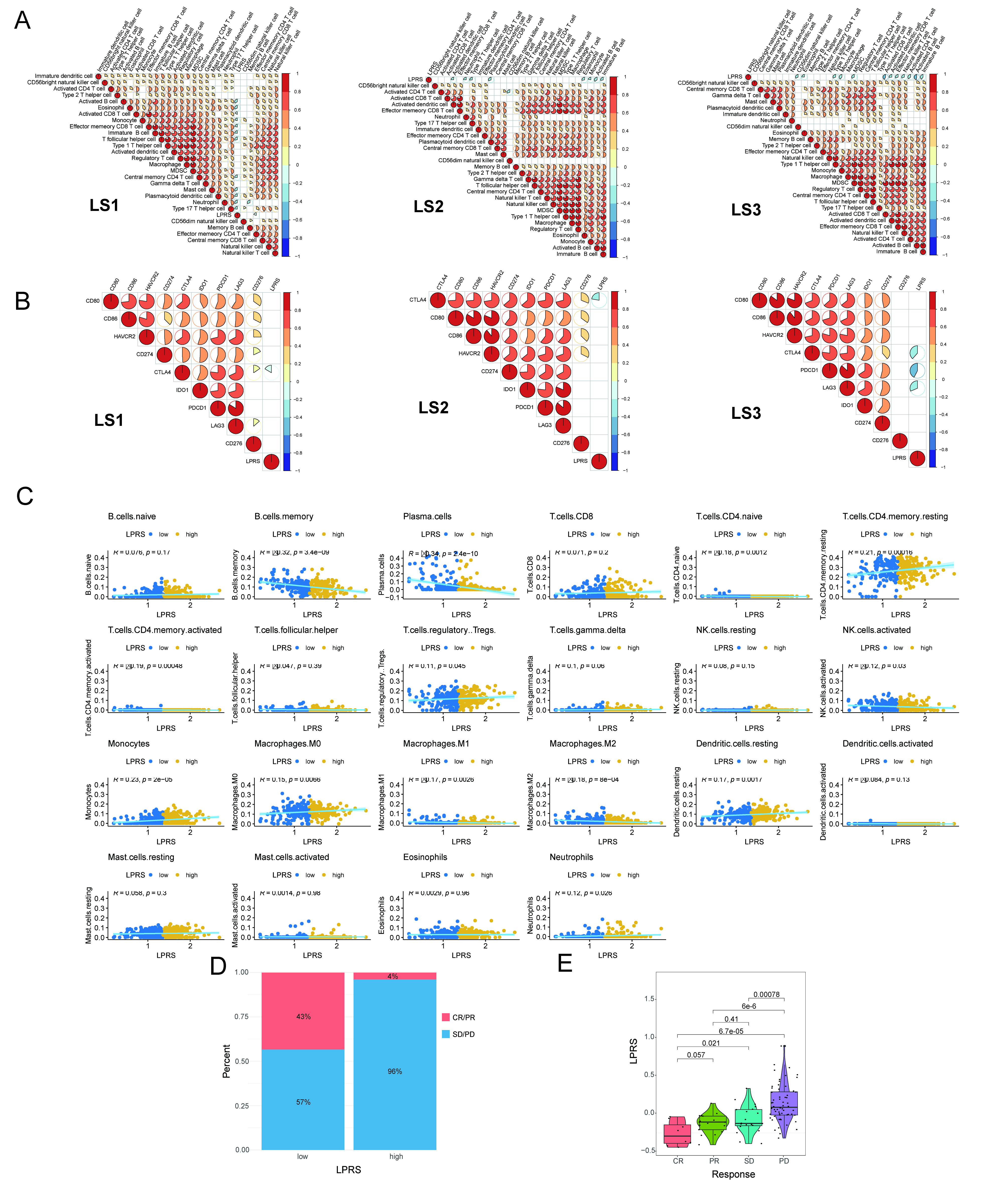


**Fig. S4 Correlation analysis between LPRS and TIME and ICI prediction.** **(A)** Correlation Between LPRS and ssGSEA Z-scores of 28 Immune Features Across LLPS Subtypes. **(B)** Expression of the Correlation Between LPRS and Immune Checkpoints Across LLPS Subtypes. **(C)** Correlation analysis. Correlation analysis between 22 immune pathways quantified by LPRS and CIBERSORT algorithms. **(D)** The proportion of patients responding to anti-PD-L1 immunotherapy in the IMvigor210 cohort (CR/PR vs. PD/SD: 4% vs. 96% in the high-LPRS subgroup, CR/PR vs. PD/SD: 43% vs. 57% in the low-LPRS subgroup). **(E)** Comparison of LPRS levels across different response categories to PD-L1 immunotherapy in the IMvigor210 cohort. CR: complete response, PR: partial response, PD: progressive disease, SD: stable disease.


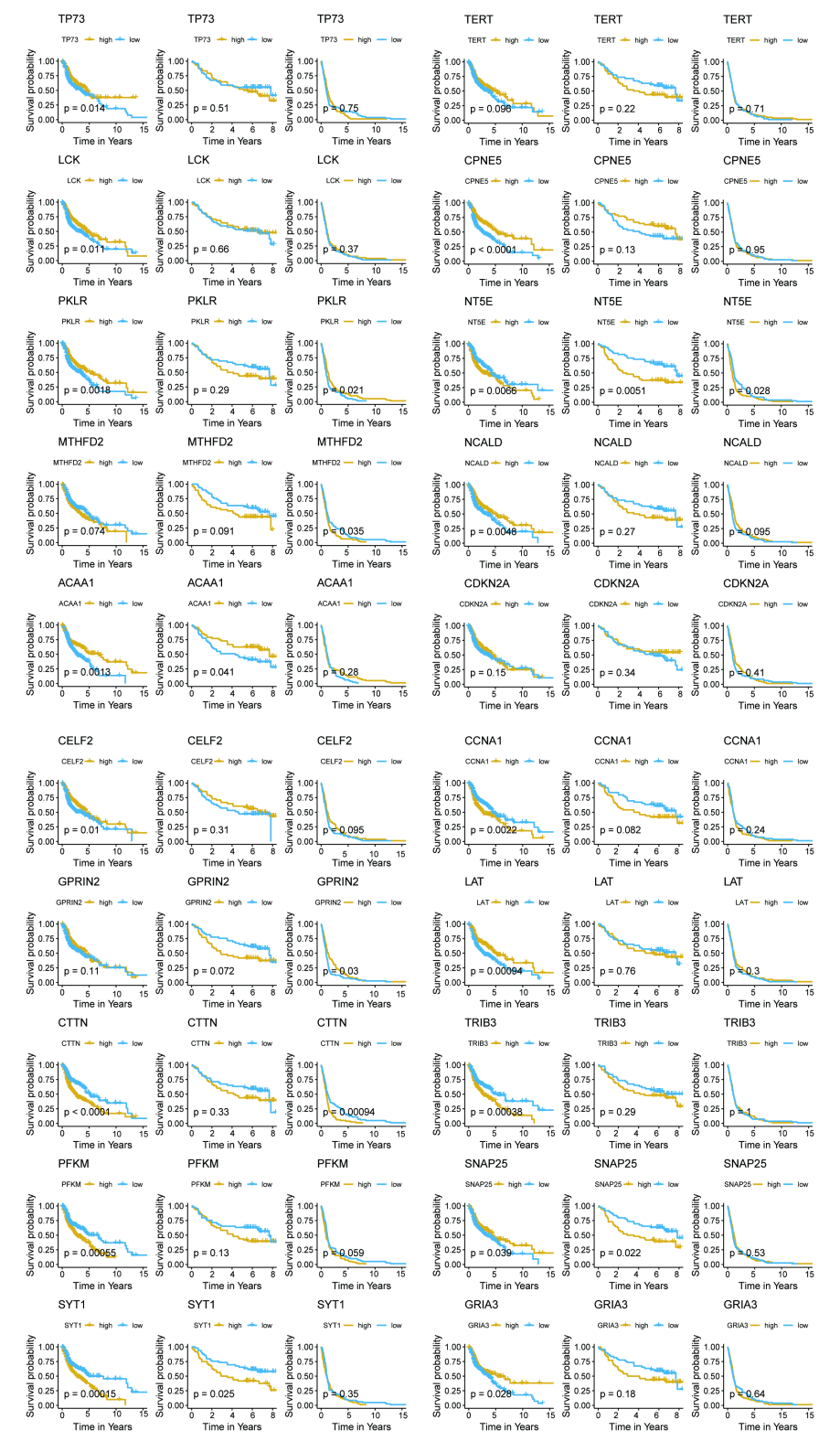


**Fig. S5** **KM plots of 20 hub genes in TCGA, GSE41613, and ICGC cohorts.** KM plots of 20 hub genes in TCGA, GSE41613, and ICGC cohorts were analyzed to identify genes with statistically significant overall survival (OS) across the three datasets.


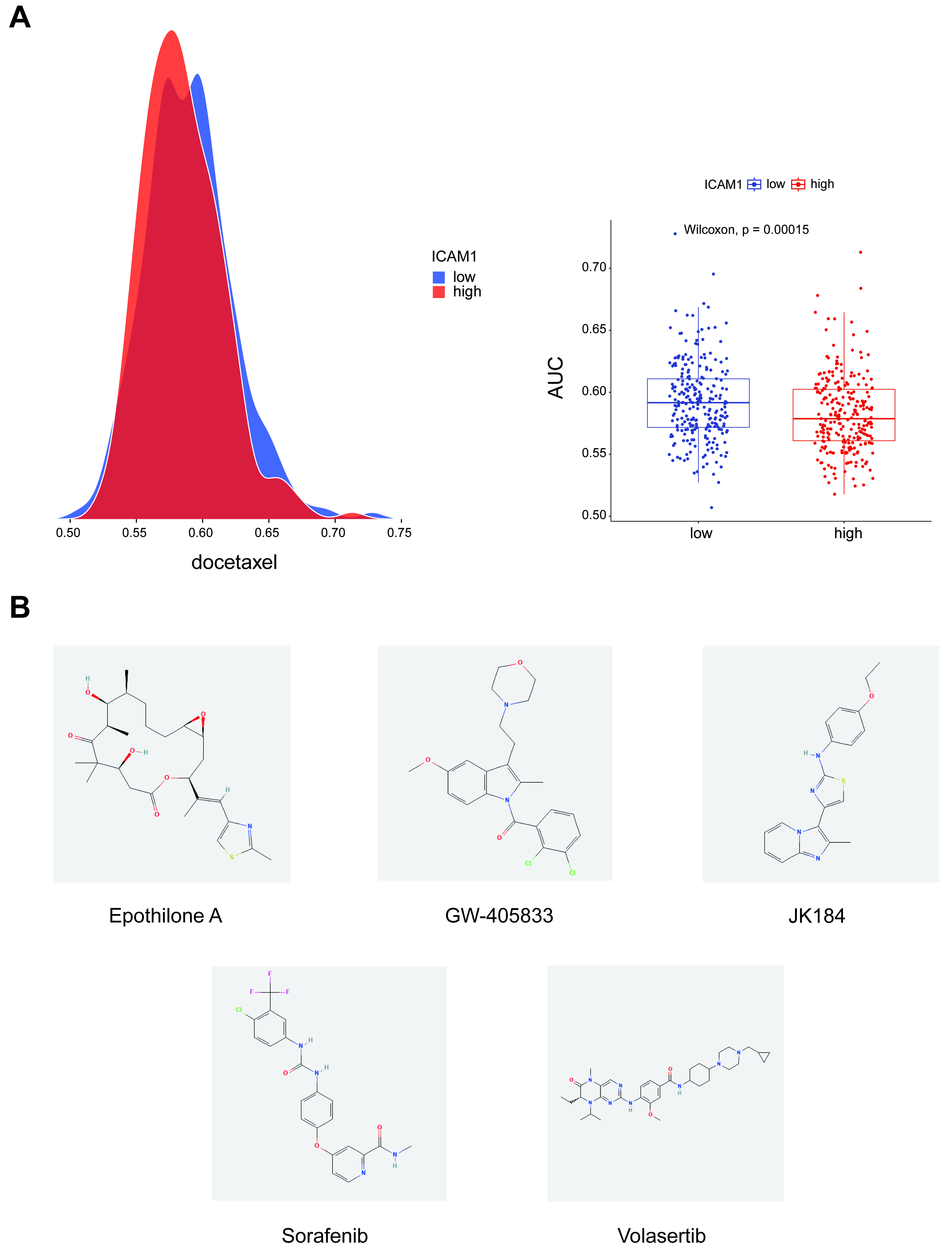


**Fig. S6** **Validation of the estimated AUC values in the TCGA-HNSC cohort, and the small molecule structures of the top five potential compounds.** (**A)** Comparison of the estimated sensitivity to docetaxel between high and low ICAM1 expression groups. (**B)** Chemical structures of the top five potential small molecule compounds.


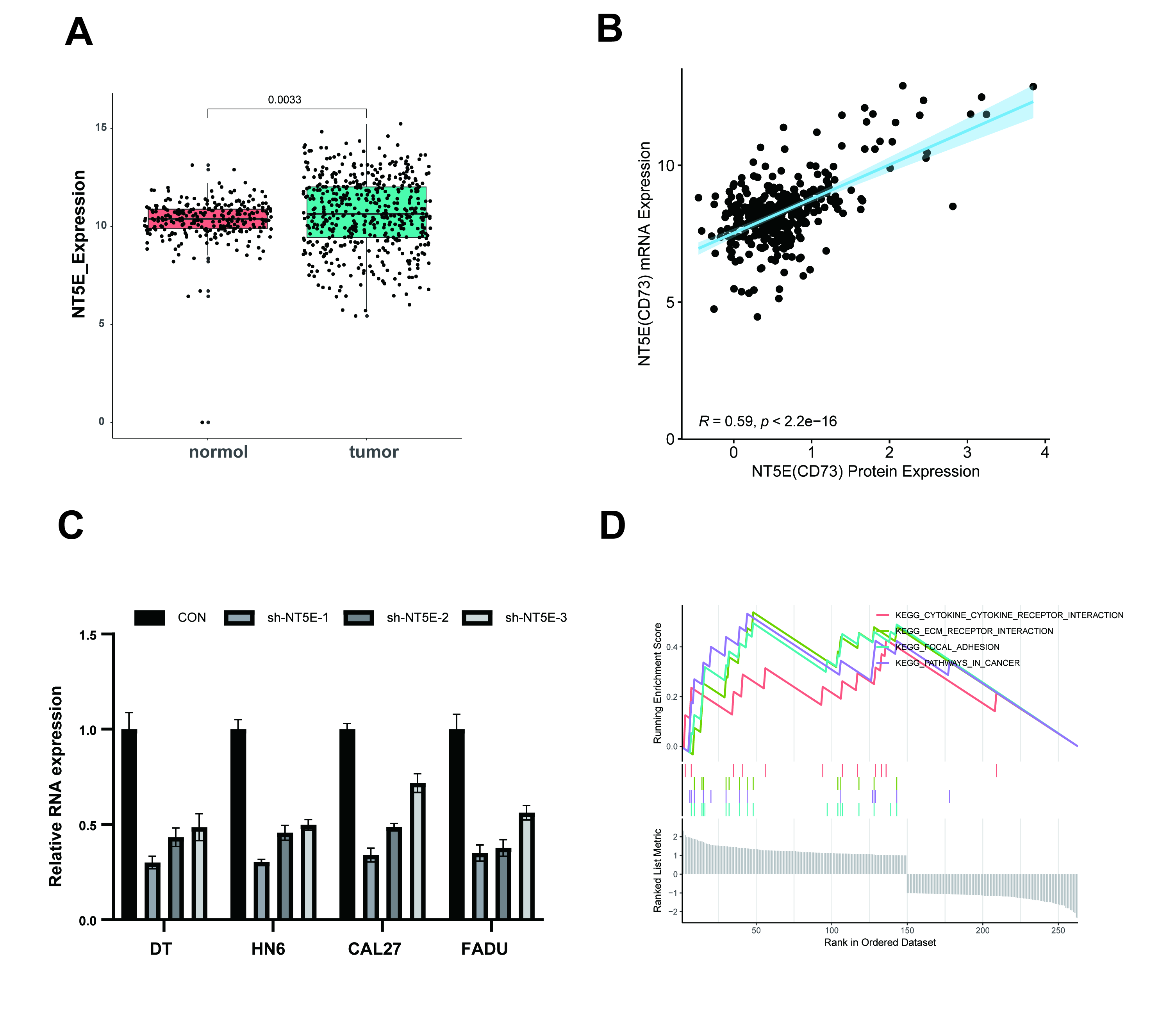


**Fig. S7** **NT5E(CD73) mRNA Expression and Plasmid Selection. (A)** Comparison of NT5E(CD73) mRNA expression levels between normal and tumor tissues in the TCGA cohort. (**B)** Correlation analysis between NT5E (CD73) protein expression and mRNA expression. **(C)** Validation of NT5E(CD73) knockdown efficiency in HNSC cell lines using qRT-PCR to assess the effectiveness of NT5E(CD73)-targeting interference fragments. **(D)** GSEA enrichment analysis of differentially expressed genes in high and low NT5E (CD73) groups

## Supplementary Tables

**Additional file: Table S1** The primers for conduction of qRT-PCR

**Additional file: Table S2** The detailed information of 3541 LLPS-related genes in homo sapiens.

**Additional file: Table S3** The LLPS-related DEGs between LGG samples and normal samples.

**Additional file: Table S4** The prognostic LLPS-related genes obtained through univariate Cox regression analysis in TCGA cohort.

**Additional file: Table S5** Molecular docking scores of screened potential compounds.
